# Direct laser writing of volumetric gradient index lenses and waveguides

**DOI:** 10.1038/s41377-020-00431-3

**Published:** 2020-12-03

**Authors:** Christian R. Ocier, Corey A. Richards, Daniel A. Bacon-Brown, Qing Ding, Raman Kumar, Tanner J. Garcia, Jorik van de Groep, Jung-Hwan Song, Austin J. Cyphersmith, Andrew Rhode, Andrea N. Perry, Alexander J. Littlefield, Jinlong Zhu, Dajie Xie, Haibo Gao, Jonah F. Messinger, Mark L. Brongersma, Kimani C. Toussaint, Lynford L. Goddard, Paul V. Braun

**Affiliations:** 1 Department of Materials Science and Engineering, University of Illinois at Urbana-Champaign, Urbana, IL USA; 2 Materials Research Laboratory, University of Illinois at Urbana-Champaign, Urbana, IL USA; 3 Beckman Institute for Advanced Science and Technology, University of Illinois at Urbana-Champaign, Urbana, IL USA; 4 Department of Electrical and Computer Engineering, University of Illinois at Urbana-Champaign, Urbana, IL USA; 5 Department of Materials Science and Engineering, Stanford University, Stanford, CA USA; 6 Carl R. Woese Institute for Genomic Biology, University of Illinois at Urbana-Champaign, Urbana, IL USA; 7 Department of Mechanical Science and Engineering, University of Illinois at Urbana-Champaign, Urbana, IL USA

**Keywords:** Polymers, Silicon photonics, Integrated optics

## Abstract

Direct laser writing (DLW) has been shown to render 3D polymeric optical components, including lenses, beam expanders, and mirrors, with submicrometer precision. However, these printed structures are limited to the refractive index and dispersive properties of the photopolymer. Here, we present the subsurface controllable refractive index via beam exposure (SCRIBE) method, a lithographic approach that enables the tuning of the refractive index over a range of greater than 0.3 by performing DLW inside photoresist-filled nanoporous silicon and silica scaffolds. Adjusting the laser exposure during printing enables 3D submicron control of the polymer infilling and thus the refractive index and chromatic dispersion. Combining SCRIBE’s unprecedented index range and 3D writing accuracy has realized the world’s smallest (15 µm diameter) spherical Luneburg lens operating at visible wavelengths. SCRIBE’s ability to tune the chromatic dispersion alongside the refractive index was leveraged to render achromatic doublets in a single printing step, eliminating the need for multiple photoresins and writing sequences. SCRIBE also has the potential to form multicomponent optics by cascading optical elements within a scaffold. As a demonstration, stacked focusing structures that generate photonic nanojets were fabricated inside porous silicon. Finally, an all-pass ring resonator was coupled to a subsurface 3D waveguide. The measured quality factor of 4600 at 1550 nm suggests the possibility of compact photonic systems with optical interconnects that traverse multiple planes. SCRIBE is uniquely suited for constructing such photonic integrated circuits due to its ability to integrate multiple optical components, including lenses and waveguides, without additional printed supports.

## 3D printing: SCRIBE tunes refractive index within optical elements

A 3D printing technique can make a tiny structure with a varying refractive index, opening the door for manufacturing lenses and waveguides with interesting properties. Subsurface controllable refractive index via beam exposure (SCRIBE), developed by Paul V. Braun and colleagues at the University of Illinois at Urbana Champaign, involves changing a laser’s power to variably fill the nanosized pores of a silicon scaffold with photosensitive polymer. This tunes the refractive index within the final, fabricated, submicron-scale structure, causing visible light to bend differently within it according to where it passes. SCRIBE was used to fabricate a 15-micrometer diameter, spherical Luneburg lens in which the refractive index decreases from its core towards the surface. SCRIBE is particularly suited for constructing multiple components, like lenses and waveguides, for photonic integrated circuits, without the need for additionally printed supports.

## Introduction

Multiphoton direct laser writing (DLW) is an emerging submicron-scale additive manufacturing technique for fabricating miniaturized three-dimensional (3D) photonic devices^1–5^. In DLW, optical components are formed with submicron voxel resolution in the photoresist by a pulsed femtosecond laser via a multiphoton polymerization process^6^. DLW has been used to form lenses^2,7–9^, mirrors^3,10^, waveguides^11^, photonic crystals^4,7,12^, phase masks^13,14^, and other related optical elements for beam shaping, imaging, and photonic integration. While DLW is now widely accessible due to advancements in instrumentation and photoresist chemistries^1,15,16^, DLW-fabricated microscale optics remain limited by the photoresist’s single refractive index^17,18^. Furthermore, the DLW process precludes the fabrication of free-standing elements, limiting the formation of compound lenses and intricate waveguiding photonic networks.

Here, we present the subsurface controllable refractive index via beam exposure (SCRIBE) method, a lithographic approach that transforms the purview of devices that can be rendered with DLW. Using SCRIBE, microscale subsurface optics, including gradient and single index lenses, compound lenses, photonic nanojet generators, waveguides, and other optical elements are generated inside the volume of thick porous silicon (PSi) and porous silicon dioxide (PSiO_2_) films. The mesoporous hosts suspend the 3D structures and stabilize the variable fill fraction of the cross-linked photoresist, enabling refractive index control over a broad range (Δ*n* > 0.3 at visible wavelengths). Changing the laser power as the beam writes enables the photoresist fill fraction (the degree of polymer infilling within the pores) and thus the effective refractive index to be spatially varied with submicron resolution.

With SCRIBE, we present the first 3D gradient refractive index (GRIN) fabrication process that attains submicron spatial and refractive index resolution. SCRIBE lithography’s parametric versatility enables geometric and GRIN configurations previously unachievable with conventional DLW. The microlenses presented in this work include doublets chromatically corrected across visible wavelengths, cascaded photonic nanojet generators, and 3D Luneburg lenses that feature spherical refractive index profiles and geometries. The 15-µm diameter spherical Luneburg GRIN lens illustrates one of the most powerful manufacturing advantages of SCRIBE in its ability to simultaneously control the geometry and refractive index in 3D space. This Luneburg lens focuses light at visible wavelengths and is the smallest spherical Luneburg lens we are aware of to this date. Furthermore, we take the first steps towards volumetric photonic integration by printing suspended 3D single-mode waveguides coupled to all-pass microring resonators. A measured loss of 2.5 dB mm^−1^ suggests SCRIBE’s potential for designing densely packed optical elements across multiple planes. SCRIBE ultimately demonstrates design versatility that complements existing multiphoton lithography approaches used for optical component fabrication.

## Results

### Broad continuous microscale refractive index control

**Fig. 1:**
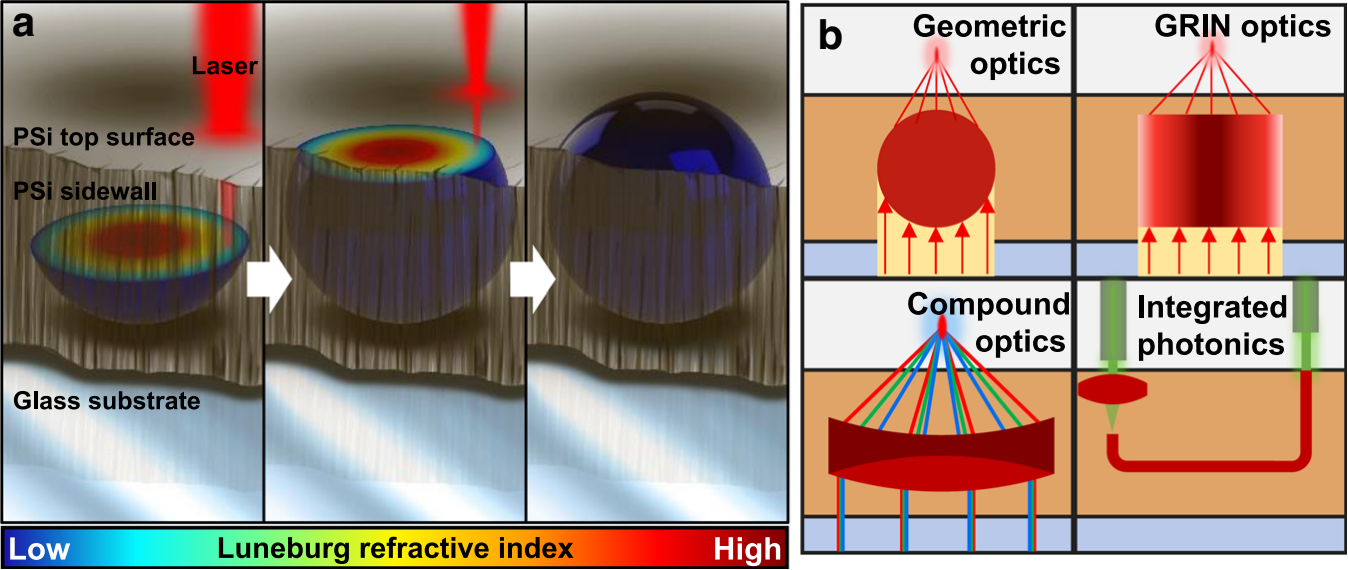
Overview of devices printed using SCRIBE. **a** Concept art showing the formation of a spherical Luneburg lens printed inside PSi with SCRIBE. **b** Schematic showing four classes of micro-optical elements printed inside a porous scaffold using SCRIBE

SCRIBE generates subsurface optical elements by focusing a pulsed femtosecond laser to locally polymerize a negative-tone photoresist inside a porous medium. The fill fraction of the polymerized photoresist inside the mesoporous scaffold is modulated by controlling the laser exposure during writing, resulting in an unprecedented tunable refractive index range of 1.28 (index of unfilled scaffold) to 1.85 for 633 nm light. Figure 1a depicts how this index range can be utilized to produce a GRIN Luneburg lens that focuses visible wavelengths. This 3D spatial control of the degree of polymer infilling allows the fabrication of geometric, compound, and GRIN optics, as well as integrated photonics (Fig. 1b).

**Fig. 2:**
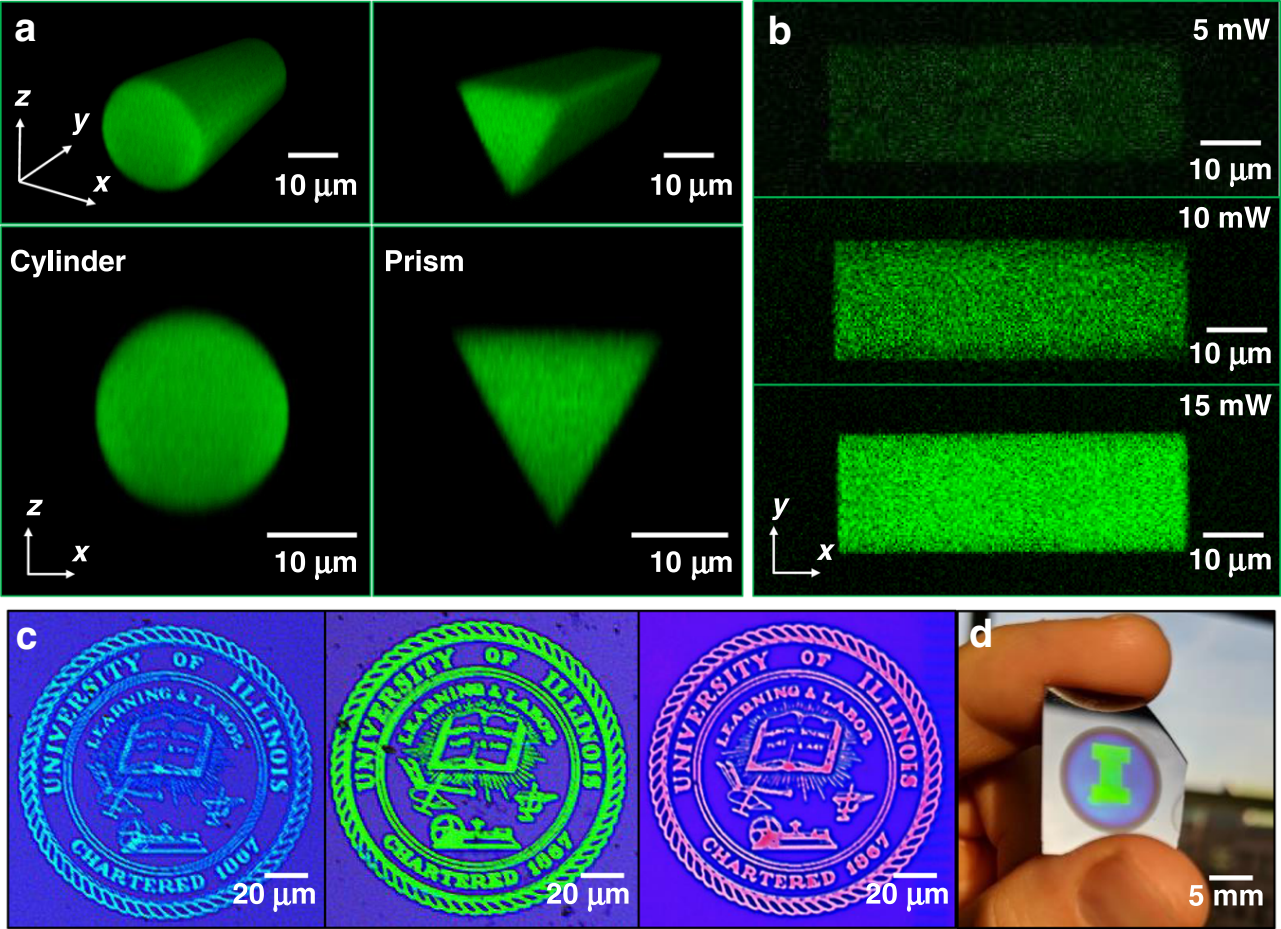
Freeform geometric and refractive index engineering with SCRIBE. **a** Multiphoton 3D and *xz*-plane fluorescence scans of a subsurface cylinder and prism. **b** Multiphoton images of three rectangular prisms printed at increasing laser exposures. Objects printed with higher laser exposures fluoresce more intensely. **c** Optical images of three University of Illinois seals printed inside blue PSi DBRs, with increasing printing laser exposure from left to right. A larger redshift in the stopband was observed as the writing exposure was increased. **d** A 5 mm × 7 mm University of Illinois “I” logo printed inside a blue PSi DBR

The geometries and variable polymer fill fractions of printed structures are experimentally observed using multiphoton fluorescence microscopy^19^. The geometric fidelity of SCRIBE is demonstrated by printing and imaging the fluorescence of simple shapes inside PSi (Fig. 2a) and is further validated by scanning electron microscopy (SEM) in the Supplementary Information (SI) Section 1. Since the fluorescence intensity is a proxy for the polymer fill fraction, the index profile of a structure can also be qualitatively determined via multiphoton microscopy. Figure 2b shows the fluorescence images of three geometrically identical prisms written with successively higher average laser powers (5, 10, and 15 mW) visualized under the same imaging conditions. The prisms written at higher laser exposures exhibited relatively higher fluorescence intensities. A checkerboard structure printed with alternating average laser powers (7.5 and 15 mW) and imaged with multiphoton microscopy, shown in SI Section 2, further demonstrates SCRIBE’s volumetrically precise control of the refractive index within a single object.

Shifts in the refractive index are visibly demonstrated by cross-linking large-area patterns inside PSi-based distributed Bragg reflectors (DBRs). The PSi DBR stopband is determined by the refractive indices and thicknesses of the alternating layers. The reflection peak is redshifted by >100 nm when the pores are infilled, with larger redshifts corresponding to higher index secondary materials^20,21^. In Fig. 2c, the submicron regions of a blue DBR (initial stopband at 495 nm) are spectrally shifted to generate 120-μm diameter University of Illinois seals including a seal redshifted to adopt the university’s orange and blue motif. As a demonstration of larger area patterning using SCRIBE, a 5 mm × 7 mm green University of Illinois block “I” was fabricated inside a blue DBR by stitching exposure fields together (Fig. 2d).

### Voxel dimension measurements

**Fig. 3:**
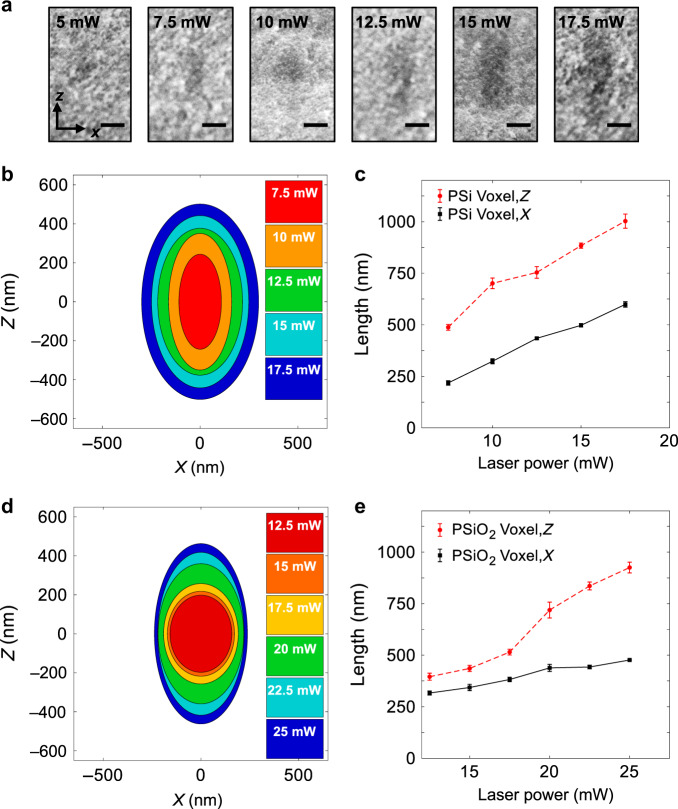
Subsurface line voxel dimensions. **a** Scanning electron microscopy of fractured cross sections of line voxels printed inside PSi at different average laser powers. The shape of the voxel becomes more asymmetric and elliptical with increasing laser power, as seen by the contrast between the polymerized region and the porous background. Scale bars are 250 nm. **b**, **d** Best-fit ellipses for the PSFs in PSi (**b**) and PSiO_2_ (**d**) in the *xz*-plane for the indicated printing laser powers. **c**, **e** The *x* and *z* dimensions of the voxels printed inside PSi (**c**) and PSiO_2_ (**e**) versus the average laser power. All line voxels are printed with a scan speed of 10 mm s^−1^

In multiphoton polymerization, voxels formed at the focal spot of a pulsed laser are the fundamental building blocks of 3D printed objects. The voxel’s shape is determined by the asymmetric point spread function (PSF) and thus are ellipsoids that are larger in the axial (*z*) direction^22^. The dimensions of voxels formed using DLW have been previously documented for regular substrates by printing lines and measuring their *x* and *z* dimensions^23^. However, the PSF is altered when the laser is focused inside a porous scaffold. By writing lines inside PSi and PSiO_2_, cleaving the samples, and viewing the fractured cross sections, the lateral and axial dimensions of the subsurface line voxels can be determined. Scanning electron micrographs of selected line voxels embedded in PSi are shown in Fig. 3a. The line voxels’ *x* and *z* dimensions for different exposure powers are measured from the contrast between the native scaffold and line arrays. All line voxels were written at a scan speed of 10 mm s^−1^.

Line voxels written in both scaffolds show an increase in size in both the *x* and *z* dimensions with increasing laser exposure, represented by the best-fit ellipse diagrams in Fig. 3b for PSi and Fig. 3d for PSiO_2_. The best-fit ellipses were found by measuring the dimensions of 20 line voxels printed at each laser power. The trends in the scaling of the voxel size with respect to laser exposure are graphically shown in the elliptical contour plots, where the central red voxel represents the lowest exposure conditions needed for observable cross-linking, and the outer dark blue voxel represents the exposure condition before the pulsed laser ablates the photoresist. The average dimensions of the line voxels printed inside both materials are also graphically depicted in Fig. 3c and Fig. 3e for PSi and PSiO_2_, respectively. With the voxel dimensions quantified with respect to laser power, 3D structures can be fabricated with precise geometries.

### Refractive index characterization

**Fig. 4:**
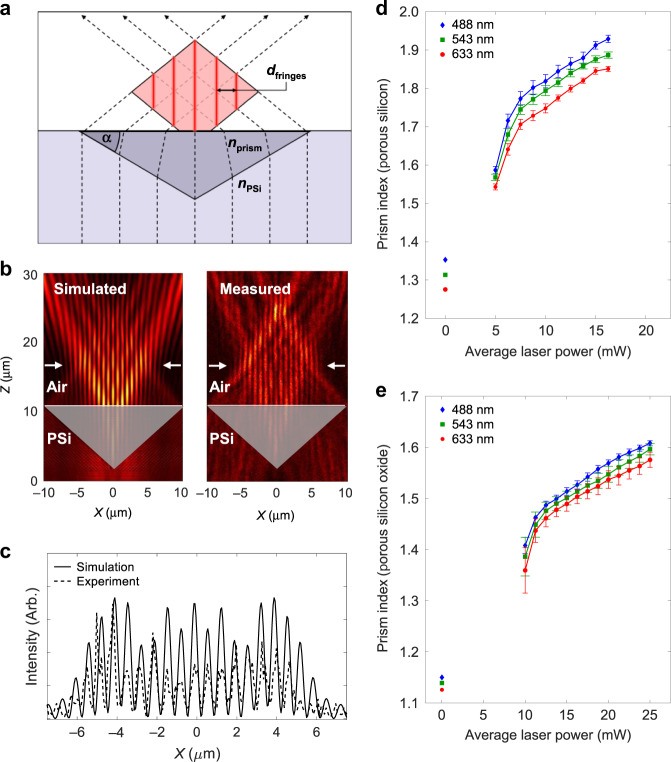
Characterizing the refractive index as a function of the writing laser power. **a** Schematic of an interference pattern produced by a subsurface Fresnel biprism. **b** The *xz*-plane cross section of the simulated and measured interference patterns at 633 nm produced by a Fresnel biprism with an effective refractive index of 1.82. **c** Overlaid intensity profiles of the simulated and measured interference patterns at the cut line shown in **b**. **d**, **e** Plots of the refractive index versus average laser power of prisms written inside PSi (**d**) and PSiO_2_ (**e**) for blue (488 nm), green (543 nm), and red (633 nm) light. All biprisms were printed with a scan speed of 10 mm s^−1^

The effective refractive index of features formed via SCRIBE as a function of laser write power was determined by writing and characterizing subsurface Fresnel biprisms inside PSi and PSiO_2_ (Fig. 4). A Fresnel biprism refracts light, forming a periodic interference pattern at the output^24,25^, as depicted in Fig. 4a. We illuminate the embedded biprisms with a laser and measure the resulting fringe spacing in air. For this configuration, the effective refractive index of the SCRIBE-rendered biprism (*n*_prism_) was derived using existing equations relevant to biprisms in the literature^24^:

1 $$n_{\mathrm{prism}} = \sqrt {n_{\mathrm {PSi}}^2\, {\rm{sin}}^2\alpha +\frac{\left[ \frac{\lambda _{\mathrm {laser}}}{2d_{\mathrm {fringes}}} + \frac{n_{\mathrm {PSi}}\,{\rm{sin}} \left( {2\alpha }\right)}{2} \right]^2}{{\rm{sin}}^2\alpha}}$$

where *d*_fringes_ is the output fringe spacing, *λ*_laser_ is the illuminating wavelength, *n*_PSi_ is the background refractive index of the porous host (measured by ellipsometry), and *α* is the prism angle, which is defined by adjusting the prism height and width. As expected, the fringe spacing decreases with increasing laser writing power.

Equation (1) assumes that the polymerized region within the porous scaffold is optically isotropic. While this approximation is valid for PSiO_2_ scaffolds, PSi is highly birefringent. PSi etched under the conditions used for these experiments showed a birefringence of ~0.15 (ref. ^26^), with the fast axis corresponding to the optical axis. When the PSi is infilled with polymerized photoresist, the birefringence is reduced but remains present, making it necessary to verify that the isotropic approximation in Eq. (1) is valid for this scaffold. Birefringent biprisms were modelled in COMSOL Wave Optics with varying ordinary (*n*_o_) and extraordinary (*n*_e_) refractive indices. The simulated biprisms were illuminated as described in Fig. 4a, and *d*_fringes_ were measured. The simulated fringe spacing consistently matches the fringe spacing calculated using Eq. (1) given that *n*_o_ used in the simulation equals *n*_prism_ used in the equation. The illuminating wavevector is mostly parallel to the optical axis if the refraction angle is small, so for PSi *n*_prism_ = *n*_o_ when *α* is small (*α* ≤ 50°). Similarly, for the other optical elements in this work that are embedded in PSi scaffolds, the angle of refraction is sufficiently small such that Eq. (1) is valid. Further discussion on these simulations is available in SI Section 3.

Figure 4b depicts simulated and measured fringe patterns for a prism with *n*_prism_ = 1.82 embedded inside PSi with *n*_PSi_ = 1.28. The *xz*-plane intensity profile of the biprism was recorded using a confocal microscope under 633 nm plane wave illumination. The line cut comparing the interference fringe pattern produced by the simulated and fabricated devices in Fig. 4c confirms a match between measurement and simulation.

Wavelengths of 488, 543, and 633 nm were used to capture *n*_prism_ for a range of average laser writing powers. Figure 4d, e shows the refractive index as a function of writing power at these wavelengths for biprisms written in PSi and PSiO_2_. Error bars are the standard error across 10 different prisms for each wavelength and average writing power. The plotted ranges of refractive index at 633 nm accessible using SCRIBE are 1.54–1.85 and 1.36–1.58 for features written with a scan speed of 10 mm s^−1^ inside PSi and PSiO_2_, respectively. The bounds of the range of plotted continuous index tuning are set by the threshold laser polymerization power at which prisms with good geometric fidelity were formed (lower bound) and the laser-induced damage limit (upper bound), as further explained in SI Section 4. The refractive index can be lower than the minimum indices plotted in Fig. 4d, e, but index measurements using these extremely low index prisms were unreliable. The data graphed in Fig. 4d, e are extrapolated when designing optical elements such as GRIN lenses that require a higher index difference than 0.3. The refractive index of the host material provides the background index for the cross-linked geometries, which are 1.28 (PSi) and 1.13 (PSiO_2_) at 633 nm.

### Lenses with chromatic dispersion control

Lenses made from dispersive materials exhibit chromatic aberration, which is typically corrected by combining lenses with different dispersions and curvatures into compound optical elements^27^. Lenses formed inside PSi are dispersive (Fig. 4d) and are therefore expected to exhibit wavelength-dependent focusing. The highly dispersive regions of unfilled PSi above and below the lens act as additional lenses with complementary dispersions and geometries. As shown here, the net result is that the chromatic focusing behaviour of lenses formed via SCRIBE differs considerably from lenses printed in air. The surrounding PSi alters the chromatic aberration curve, transforming a dispersive singlet into an achromat.

**Fig. 5:**
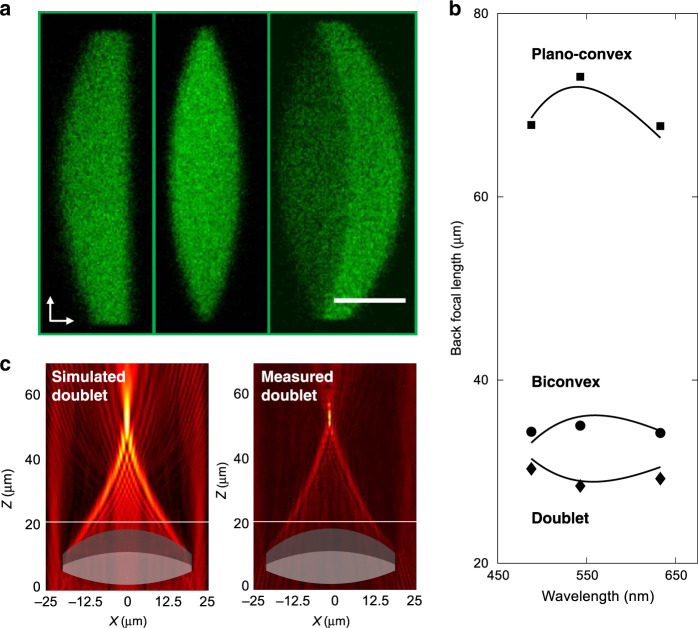
Dispersion control in geometric optics. **a** Multiphoton cross-sectional images of microscale planoconvex, biconvex, and achromatic doublet lenses written inside PSi. Each of the doublet’s components are rendered using different average laser powers, as indicated by the different fluorescence intensities. **b** Measured focal lengths of the planoconvex (square), biconvex (circle), and doublet (diamond) lenses shown in **a** when illuminated at 488, 543, and 633 nm. Solid traces depict the simulated (Zemax) focal lengths of the elements versus the wavelength. **c** Simulated and measured focal behaviour for the doublet shown in **a** at 633 nm

Three 40 µm diameter lenses, namely, a planoconvex singlet, a biconvex singlet, and a Fraunhofer doublet, were fabricated inside PSi, as visualized in Fig. 5a. The cross sections are imaged using multiphoton microscopy, where the fluorescence intensity corresponds to the fill fraction of the polymerized photoresin. This effect is most evident in the doublet where a change in the fluorescence intensity marks the cross-sectional outline. The design parameters of each lens are outlined in Table 1, including the radius of curvature (ROC), thickness, lens material, and Abbe number of each region. The dispersive characteristics of the different printed regions account for the chromatic behaviour observed for these elements.

**Table 1:** Parameters for the chromatically corrected lenses

	Surface	ROC (µm)	Thickness (µm)	Material	Abbe number
Plano convex	1	∞	15	PSi	3.8
	2	50	10	PSi/PR (12.5 mW)	13.0
Biconvex	1	∞	12.5	PSi	3.8
	2	50	10	PSi/PR (12.5 mW)	13.0
	3	−50	2.5	PSi	3.8
Fraunhofer doublet	1	∞	5.5	PSi	3.8
	2	50	10	PSi/PR (7.5 mW)	11.1
	3	−50	7	PSi/PR (12.5 mW)	13.0
	4	−30	2.5	PSi	3.8

The PSi surrounding each lens acts like a low index highly dispersive optical medium since its Abbe number is always lower than the polymerized regions; this effectively transforms a planoconvex lens printed inside PSi at the surface into an achromatic doublet and a biconvex lens into a triplet with even further reduction in chromatic aberrations. Figure 5b depicts the chromatic aberration curves for all three fabricated lenses inside PSi. The curves for the planoconvex and biconvex lenses show achromatic focusing behaviour, with the focal length at 488 nm matching that at 633 nm in both cases. The additional curved surface and the additional PSi region above the biconvex lens reduce the chromatic focal shift from 5 µm for the planoconvex lens to 0.8 µm for the biconvex lens.

The varying Abbe number with writing power underscores SCRIBE’s potential to engineer the chromatic focusing of multicomponent lenses. As a demonstration, a Fraunhofer doublet was formed inside PSi where each lens was printed at a different power. By engineering the ROCs and Abbe numbers of the two lenses, the Fraunhofer doublet was designed to reverse the sign of the concavity of its chromatic aberration curve while maintaining a small chromatic focal shift (~1.87 µm). This effect is highlighted in Fig. 5b, which shows how the element’s focal length is shorter at 543 nm than at 488 and 633 nm. A focal profile comparison (Fig. 5c) shows close agreement between the measured and simulated back focal length and numerical aperture (NA), 32 μm and 0.44 (simulation) and 31 μm and 0.43 (measurement).

### Stacked multicomponent interference-based optics

The Fraunhofer doublet (Fig. 5) highlights the scaffolding medium’s ability to simplify the fabrication of multicomponent devices by removing the need for additional alignment and support structures. As a further exploration of stacked multicomponent elements, we fabricated cascaded multimode interference microstructures (SI Section 5). This device design was previously proposed for generating photonic nanojets^28^, near subwavelength foci that propagate over distances greater than a wavelength^29^.

Unlike photonic nanojets generated by dielectric microspheres and microcylinders^30,31^, the proposed cascaded device features additional geometric parameters (Fig. S6a) that lead to greater control over the output beam’s characteristics. This design specifically uses engineered modal interference to suppress the sidelobes of the output beam, producing a cleaner and narrower near diffraction-limited hotspot than that produced by a singlet (Fig. S6c, d).

### Planar and 3D GRIN lenses

**Fig. 6:**
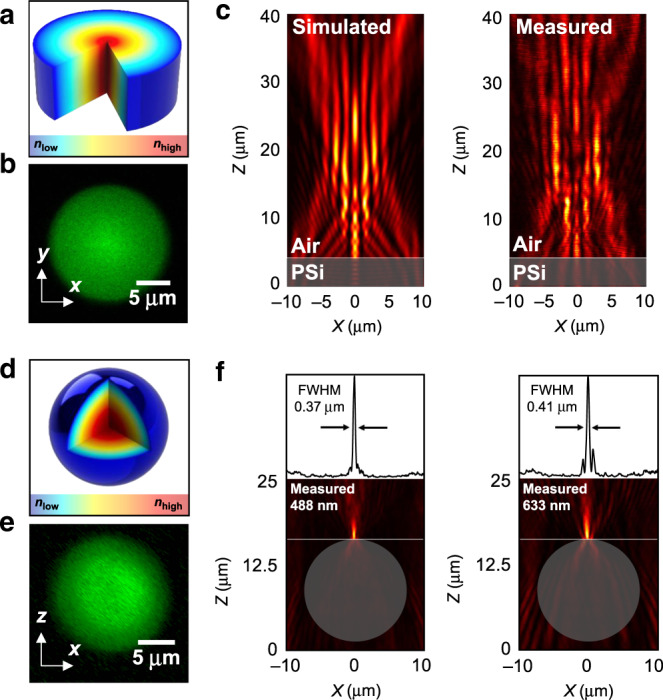
Gradient refractive index optical elements. **a**, **d** 3D cutaway diagrams of a GRIN axicon (**a**) and a spherical Luneburg lens (**d**). **b**, **e** Measured multiphoton fluorescence images of a GRIN axicon (*xy*-plane) (**b**) and the spherical midsection of a Luneburg lens (*xz*-plane) (**e**) printed in PSi. **c** Simulated and measured *xz*-plane intensity profiles of interference patterns produced by a GRIN axicon printed in PSi focusing 633 nm light. **f** Measured *xz*-plane intensity profiles of Luneburg lenses printed in PSi focusing 488 and 633 nm light on their surfaces, with FWHMs of 0.37 and 0.41, respectively

GRIN lenses have long been proposed as an alternative to geometric optics due to their ability to reduce geometric aberrations^32^. A common GRIN optic is a planar lens with a radially varying refractive index^33^. SCRIBE was used to construct such a 20-μm diameter flat axicon by radially modulating the laser exposure such that the index profile follows:

2 $$\begin{array}{*{20}{c}} {n\left( r \right) = n_{{\mathrm {centre}}} - \frac{{n_{{\mathrm {centre}}} - n_{{\mathrm {edge}}}}}{{R_{{\mathrm {lens}}}}} \ast r} \end{array}$$

where *n*_centre_ and *n*_edge_ were set at 1.8 and 1.6 for 633 nm light, respectively. The planar axicon’s index distribution is depicted in Fig. 6a and was visualized using multiphoton fluorescence microscopy (Fig. 6b), where the peak fluorescence intensity at its centre corresponds to the region of the highest refractive index.

This planar axicon outputs a Bessel-like beam with a ring-shaped intensity distribution (SI Section 6) when illuminated with a plane wave. The cross section of the device’s output agrees with the simulation in Fig. 6c, verifying SCRIBE’s ability to accurately define the refractive index in the planar optical elements. Various other flat elements that function as parabolic lenses or cubic Airy beam phase masks can be generated by altering *n*(*r*) in Eq. (2) (SI Section 7). As we now discuss, these same design principles can be extended to three dimensions to form spherical GRIN lenses and other volumetric elements with arbitrary geometries and index profiles.

A Luneburg lens is an aberration and coma-free lens with a spherically symmetric refractive index profile (Fig. 6d) that follows Rudolf Luneburg’s solution^34^:

3 $$\begin{array}{*{20}{c}} {n_{{\mathrm {Luneburg}}} = \sqrt {2 - \left( {\frac{r}{{R_{{\mathrm {lens}}}}}} \right)^2} } \end{array}$$

A Luneburg lens is a unique GRIN element that focuses incident rays on its opposite surface in a manner unobtainable in spherical homogeneous refractive index lenses. To date, the smallest Luneburg lenses have been manufactured with DLW by structuring 3D metamaterials containing unit cells with gradient volumetric variations in the polymer and air^35^. However, the unit cell sizes achievable with the resolution of DLW restrict the devices’ operation to infrared wavelengths. Prior to this work, no fabrication technique has yielded a spherical Luneburg lens that focuses visible light.

SCRIBE’s ability to spatially modulate the refractive index within a 3D geometry provides the basis for a visible wavelength Luneburg lens. A 15-µm diameter spherical Luneburg lens was realized by rastering the laser exposure volumetrically inside the element. The refractive index profile of this Luneburg lens is equivalent to Eq. (3) but offset by the refractive index at the surface of the lens:

4 $$\begin{array}{*{20}{c}} {n_{\mathrm {Luneburg}} = n_{\mathrm {surface}} - n_{\mathrm {air}} + \sqrt {2 - \left( {\frac{r}{{R_{\mathrm {lens}}}}} \right)^2} } \end{array}$$

The GRIN profile of the lens was visualized by capturing a multiphoton fluorescence image at the spherical midsection of the printed lens (Fig. 6e), where the gradient index is represented by a gradual change in the fluorescence intensity from the centre to edge.

Luneburg lenses were designed and characterized at different wavelengths. Figure 6f shows the cross sections of the 15-µm diameter spherical Luneburg lenses focusing 488 and 633 nm light on their opposite surfaces (top view in SI Section 6). As with any Luneburg lens, the NA is measured to be 0.707. The resolution-limited measured full-width at half-maximum (FWHM) was 0.37 and 0.41 μm at 488 and 633 nm, respectively.

### 3D waveguides and integrated photonics

**Fig. 7:**
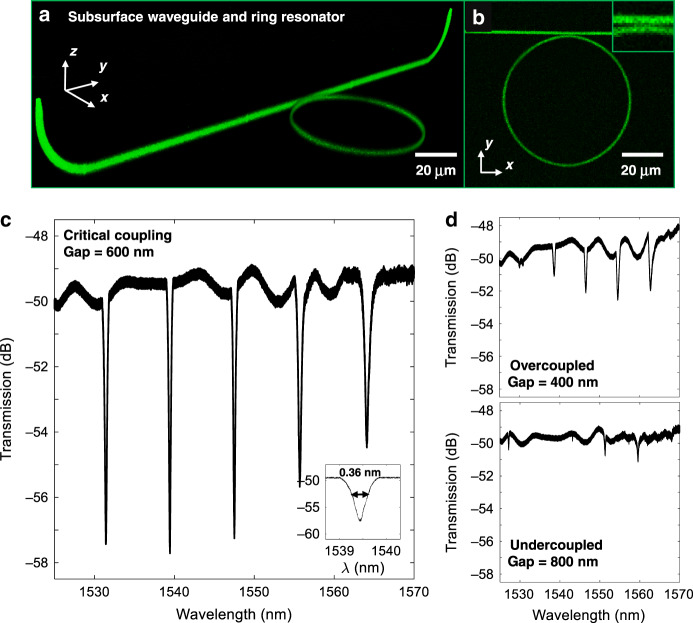
3D GRIN waveguides coupled to ring resonators. **a** 3D multiphoton imaging of a subsurface U-shaped waveguide printed in PSiO_2_ coupled to a microring resonator, with coupling ports displaced by 250 μm. **b** Top-view multiphoton image of a 60-μm diameter all-pass microring resonator coupled to a bus waveguide. The inset shows the submicron gap between the microring and the bus waveguide. **c** Measured microring resonator spectrum for a 600 nm gap device operating in TE mode (inset: resonance near 1539.5 nm shows a 0.36 nm FWHM). **d** Microring spectrum for a 400 and 800 nm gap device operating in TE mode

A unique aspect of SCRIBE is its ability to define fibre-like structures across three dimensions, enabling the formation of multiplanar waveguides. A U-shaped GRIN waveguide coupled to a ring resonator was printed to demonstrate the 3D routing of light and to enable quantitative measurements of the optical propagation loss. Figure 7 shows the U-shaped subsurface 3D waveguide printed in PSiO_2_, where both ends curve upwards and terminate at the top surface. The waveguide accepts light from an input fibre above the surface, guides the light through a 90° bend and straight bus waveguide, couples it to an all-pass microring resonator lying in a plane parallel to the surface, and finally turns the light 90° back up to the surface for collection by an output fibre. The 1-µm diameter single-mode GRIN waveguide was designed and fabricated, considering the elliptical PSF of the writing process, as described in SI Section 8, where single index 1-µm diameter waveguides are also discussed.

Figure 7a shows a 3D multiphoton microscopy reconstruction of the integrated device, which consists of a subsurface 3D waveguide and a 60-µm diameter microring resonator with the same cross-sectional GRIN profile as the waveguide (top view in Fig. 7b). The submicron gap between the microring resonator and the 3D bus waveguide is visible in the image. The gap was varied in 100 nm increments to determine the critical coupling condition (a detailed discussion of the device design is included in the methods section).

The transmission spectrum for the device with a 600 nm gap between the waveguide and ring resonator is graphed in Fig. 7c. This device is close to being critically coupled. The measured microring resonances are superimposed on top of the Fabry–Perot oscillations formed by the cavity of the bus waveguide with the input and output ports acting as two facets. The inset in Fig. 7c shows the dip near 1539.5 nm from which an FWHM of 0.36 nm and a *Q* of 4310 are extracted. The dip near 1547.5 nm has an FWHM of 0.33 nm and a *Q* of 4630. Figure 7d shows the transmission spectra for the 400 and 800 nm gaps, corresponding to over- and under-coupling, respectively. A rigorous loss analysis is included in SI Section 9, where the observed resonances are used to de-embed the fibre-to-waveguide coupling losses and thereby extract the propagation loss coefficient *α* and the transmission coefficient *t*_d_ using Eq. (S3)^36^. The propagation loss includes the radiation loss due to the ring’s curvature, the scattering loss due to the mesoporous scaffold, and the absorption loss from the waveguide materials. The loss for the subsurface GRIN waveguide is estimated to be 2.5 dB mm^−1^ (the loss estimate for the single index waveguides is 3.3 dB mm^−1^, SI Section 8). It is not surprising that these values are noticeably greater than the 0.72 and 0.23 dB mm^−1^ losses for step-index 60-µm diameter microrings, with a rectangular cross section, made with lithium niobate and silicon nitride cores, respectively, because those systems use electron beam lithography to minimize the scattering loss from the sidewall roughness and leverage a slightly higher core-cladding index contrast (0.77 and 0.53, respectively, versus ~0.4 here) to maximize mode confinement and minimize bending loss^37,38^. Nevertheless, we suggest that these loss and *Q* values are adequate for making small aerial footprint networks of waveguides for routing optical signals in 3D and filters for dense wavelength division multiplexing.

## Discussion

We conclude with a discussion on the utility of the SCRIBE lithographic approach for the 3D microfabrication of optical elements. SCRIBE lithography combines the geometric fidelity of DLW with an effective medium materials platform to engineer new classes of optics with unprecedented spatial control over the refractive index. With SCRIBE, porous materials such as PSi and PSiO_2_ can be partially or fully filled with a polymer by varying the exposure conditions, enabling the broad tuning of the refractive index in 3D objects. Furthermore, the far subwavelength size of the pores (averaging 60 nm in diameter) limits light scattering at visible and infrared frequencies. SCRIBE’s ability to simultaneously define volumetric shapes and index in 3D are attributes not possible via 2D patterning approaches. SCRIBE enabled the formation of the first visible wavelength 3D Luneburg lens and is also capable of realizing multicomponent compound lenses that control chromatic dispersion and that generate photonic nanojets, as well as 3D waveguides that couple light across multiple dimensions.

We suggest that GRIN lenses made using SCRIBE lithography merit consideration as wavefront-shaping devices in compact imaging systems. SCRIBE achieves an index range of 0.3 or greater across visible wavelengths and can be arbitrarily modulated, enabling flat gradient index microlenses. By arranging flat microscale lenslets into densely packed arrays, applications such as spherical aberration-free light field microscopy may be realized^39^. Furthermore, cascaded multilevel lenses with different form factors can be generated by SCRIBE in a manner that parallels the versatility and small footprint of metalenses. Additional applications we envision are interfacing with CMOS sensors, collimation, and diffusers for laser systems such as vertical-cavity surface-emitting lasers.

Single-mode waveguides were demonstrated in three dimensions using SCRIBE, suggesting the possibility of vertical photonic integration. 3D waveguides have previously been demonstrated by altering the local refractive index of glass and silicon using a femtosecond pulsed laser^40,41^. While the SCRIBE-generated waveguides have higher loss than these and conventional waveguides, SCRIBE’s ability to define waveguides with high index contrast and to integrate a diverse set of optics makes it uniquely suitable for short-range optical interconnects that traverse multiple planes.

In conclusion, we have demonstrated a new approach referred to as SCRIBE for the fabrication of optical elements by using a scaffold that supports structures containing variable photoresist fill fractions. Our approach offers submicron resolution over an unprecedented 3D refractive index range of more than 0.3, which has allowed the fabrication of a variety of optical elements, including the world’s smallest Luneburg lens. We believe this new approach will inspire an abundance of research not only for the integration of SCRIBE into other technologies but also the exploration of new materials systems that can improve upon some of the shortcomings of the materials used here. For example, the development of new photoresists that have high resolution and low fluorescence will enable bioimaging applications. The design of high refractive index photoresists will expand the tunability of optics printed with SCRIBE. Increasing the current maximum achievable index contrast from 0.57 to 1.0 could allow for the fabrication of unique optics such as Maxwell fisheye lenses, Eaton lenses, and electromagnetic blackholes. The investigation of new scaffolding materials with transparency at different wavelengths and lower losses will broaden the potential application space.

## Materials and methods

### PSi etching

Diced silicon wafers (Prolog, boron doped, 0.001 Ω cm resistivity, (100)-orientation double side polished, 500-μm-thick) were rinsed with acetone, deionized water, and isopropyl alcohol, and dried with nitrogen. The silicon chips were placed in a polypropylene cell and sealed with an O-ring that exposed a circle with an area of 1.23 cm^2^. The back side of the silicon was in contact with a stainless steel electrode. The silicon was submerged in an electrolyte with a 1:1 volume ratio of ethanol and aqueous 48% hydrofluoric acid. A counter electrode made of a 5-mm diameter platinum–iridium inoculating loop was submerged in the electrolyte 2.5 cm above the silicon. An SP-200 potentiostat delivered current to the wafer at a current density of 400 mA cm^−2^, porosifying the silicon in the exposed region. The applied current density was alternated between 400 and 300 mA cm^−2^ during DBR fabrication. After the silicon was porosified, the HF electrolyte was carefully removed from the cell, and the silicon wafer was rinsed thoroughly with ethanol. Samples that operate in a transmission mode were transferred to transmissive substrates such as fused silica or alumina. To accomplish this, PSi was electrochemically detached from the silicon chip by electropolishing the film under a high current density (400 mA cm^−2^) in an electrolyte composed of a 5:1 volume ratio of ethanol to aqueous 48 wt% hydrofluoric acid. The free-standing PSi membranes were then transferred onto these substrates using a gentle stream of ethanol.

### Thermal oxidation

PSi was converted into PSiO_2_ by thermal oxidation. The oxidation was carried out in a Lindberg Heavy-Duty Lancer M-300 oxidation tube with mass flow control of the gases. The PSi films, prepared either as on-silicon chips or transferred samples, were introduced into the furnace at a temperature of 400 °C in a nitrogen environment. The temperature was slowly increased, and when the furnace temperature stabilized at 900 °C, the N_2_ gas flow was switched off, and dry O_2_ gas was introduced into the furnace at a flow rate of 8 slm. After 30 min of oxidation, the O_2_ gas flow was terminated, and N_2_ gas flow was reintroduced during cooling. When the furnace reached a temperature of 500 °C, the sample was removed from the furnace to be used for SCRIBE fabrication.

### SCRIBE lithography

All subsurface structures were fabricated using a Nanoscribe Photonic Professional GT microscale 3D printer equipped with a femtosecond‐pulsed (100 fs, 80 MHz, 50 mW avg, 6.25 kW peak) fibre laser (FemtoFiber Pro, Toptica Photonics) with its operating wavelength centred at 780 nm and a high numerical‐aperture index-matched immersion objective (×63, NA = 1.4 or ×25, NA = 0.8). The laser repetition rate was fixed and, therefore, the peak power varied proportionally with the set average power. Geometric micro-optic and gradient index elements were designed using computer-aided design software (MATLAB, AutoCAD, and SOLIDWORKS) and input into the Nanoscribe DeScribe rendering software interface. The rendering files for all optical elements were programmed to use Nanoscribe’s galvanometric scanning mechanism to define the lateral features and the piezo actuator to structure the objects axially, allowing the fast, layer-by-layer construction of 3D architectures.

PSi and PSiO_2_ were selected as the porous scaffolds due to their low absorption coefficients (SI Section 10) at the pulsed laser source’s excitation wavelength. The porous films were infilled by dripping a commercially available negative-tone photoresist ((2‐(hydroxymethyl)‐2‐[[(1‐oxoallyl)oxy]methyl]‐1,3‐propanediyl diacrylate, known as IP-Dip) onto the porous films under vacuum. The two-photon polymerization of subsurface structures was carried out by positioning the laser focal spot within the volume of the PSi/photoresist region, and objects were formed at a writing speed of 10 mm s^−1^, with the galvanometric mirror acceleration set to 10^6^ V s^−^^2^, resulting in a writing acceleration of 47,100 mm s^−2^ and 118,000 mm s^−2^ for the ×63 and ×25 objective, respectively. A spacing of 100 nm was used between the printed line voxels. The laser exposure delivered during the writing process was altered to modify the degree of polymer filling (the polymer ‘fill fraction’) inside the porous scaffold. 3D architectures that are laterally larger than the writing field of view were stitched by dividing the writing process between multiple unit blocks split from the originally designed structure. The samples were developed in a bath of 99.5% propylene glycol monomethyl ether acetate to remove the nonpolymerized resist, rinsed in isopropanol for 1 min, and dried either supercritically or under nitrogen.

### Ellipsometry

The optical constants of the unfilled PSi and PSiO_2_ films were measured and extracted using a J.A. Woollam VASE ellipsometer. The variable angle spectroscopic ellipsometry (VASE) data and oblique incidence reflectance values were measured between 400 and 1100 nm at incident angles of 45°, 60°, and 75°. Refractive index dispersions were extracted from ellipsometric and reflectance data using a biaxial effective medium fitting model included in the J.A. Woollam VASE analysis software.

### Intensity profile confocal imaging

A WITec Alpha 300S upright confocal microscope fit with a ×100 Zeiss Epiplan-Apochromat objective (NA = 0.95) was used to perform *xz*-plane optical intensity depth scans of the SCRIBE-written optical elements’ focal behaviours. Free-space gas lasers were used for the plane wave illumination of the samples at wavelengths of 488 nm (Ar^+^), 543 nm (HeNe), and 633 nm (HeNe). Alternatively, the samples were also illuminated using a supercontinuum laser source (Fianium Supercontinuum SC450, Fianium, Southampton, UK) that was spectrally filtered by an acousto-optic tunable filter (Fianium AOTF V1, Fianium, Southampton, UK), facilitating the use of multiwavelength laser light. The beam was then spatially filtered, collimated, and directed through the sample containing the prisms, lenses, and axicons. The light was then collected by the objective. A 25-μm core diameter multimode fibre transmits the collected photons to a fibre-coupled avalanche photodiode (Micro Photon Devices). The multimode collection fibre acts as a 25-μm diameter pinhole in the confocal setup, enabling the collection of light at discrete pixels that build up the intensity scan. Confocal scanning was performed by using a piezo actuated two-axis stage with nanometric lateral resolution to image in the *x* and *y* directions and a stepper motor with 50 nm resolution to obtain the optical profile in the *z* direction. The pixels were stepped in increments of 50 nm in the *x* direction and 100 nm in the *z* direction. This process oversamples the intensity distribution and resolves the features of the intensity scan projected into the far field. Photons were collected over a 10 ms integration time, and the intensity of the light sources was adjusted with a neutral density filter to constrain the illuminating intensity within the linear range of the photon counting regime.

### Waveguide design and characterization

The bus waveguide was designed with a 1 μm diameter, and the vertical input and output ports have an interport spacing of 250 μm. The total length of the U-shaped bus was 281 μm, containing a central horizontal section measuring 213 μm in length and two vertically oriented quarter circle arcs with a radius of 18.5 μm (arc length of 29 μm) terminated with a vertical 5 μm section that couples with the characterization setup’s fibre array. A 60-μm diameter ring resonator with a core diameter of 1 μm was fabricated with the edge of the ring positioned in the middle of the bus’s horizontal central section; the gap between the bus and the ring was spaced laterally by 400, 600, and 800 nm to determine the critical coupling condition.

A near-IR tunable laser source (Photonetics Tunics Purity) driving an erbium-doped fibre amplifier was used to characterize the loss in the waveguides. An eight-channel optical fibre array (OZ Optics) was used to couple light into and out of the waveguides. The array has eight polarization maintaining optical fibres integrated in a patch cord arranged along a line with an interspacing of 250 μm. The array facet was polished at 0° to enable coupling at normal incidence. Channel 1 was used to couple light into the input port of the U-shaped waveguide, and channel 2 was used to measure the output. A manual 3-paddle polarization controller and an inline fibre polarizer were used to align the polarization to the slow axis of the input fibre and thereby excite the transverse electric mode of the microring (i.e., in-plane polarization). The transverse magnetic mode of the microring (i.e., polarization primarily aligned to the pores) was separately excited using a slow axis to fast axis adapter. The fibre array was mounted on a 1-axis goniometer to enable fine in-plane rotational alignment; the sample was placed on a rotational stage on top of an *xyz*-translational stage to enable the coarse rotational and fine translational alignment. Side-view and tilted top-view microscopes were used to aid in the alignment process. As the laser wavelength was tuned, the light signals transmitted through the device, as well as two split off fractions that pass through either a wavelength reference fibre or a plain fibre, were converted to photocurrents using fibre-coupled photodiodes, amplified with logarithmic amplifiers, and measured on an oscilloscope. The reference consists of a fibre-coupled acetylene gas cell with well-known absorption lines and several fibre Bragg gratings. This procedure enables the time-to-wavelength conversion of the oscilloscope data. The plain fibre provides a channel to measure the input power and determine the absolute transmission loss in dB. This overall setup was used to obtain the ring resonator’s transmitted intensity spectrum across the telecom C-band.

### Multiphoton imaging

Multiphoton imaging experiments were performed using a Zeiss LSM 710 NLO inverted microscope. A pulsed femtosecond Ti:Sapphire laser (Mai Tai eHP with DeepSee; Newport Corporation, Irvine, CA) with a laser excitation tunable between 690 and 1040 nm with a regular photomultiplier tube or a 32 channel quasar spectral detector was used to characterize the fluorescence emission of objects polymerized using SCRIBE. *Z*-stack acquisitions were performed with a ×63 1.4 NA oil immersion objective with a laser excitation wavelength tuned to 780 nm, giving a lateral resolution on the *x*/*y*‐axis of 265 nm and on the *z*-axis of 630 nm^42^. The average scanning laser power was set at 4%. Zeiss software’s “Auto *Z*-Correction” option in the *Z*-stack module was used during image capture to compensate for the loss in the excitation light due to absorption and scattering in the larger samples. An automated *xy*-stage and a stepper motor actuated *z*-stage were used to image the *z*-stacks of the SCRIBE-written structures at a 0.25 μm step size along the *z-*axis and a 0.1 μm pixel size in *xy*. Fields were averaged two times to increase the signal-to-noise ratio. The samples were directly immersed in Immersol index-matched oil (*n* = 1.518) during imaging. Image processing and 3D reconstruction were performed using ImageJ and Amira Software version 6.7.0.

## Supplementary information

Supplementary Information

## Data Availability

The data that support the findings of this study are available from the corresponding author upon reasonable request.
