# Supplementary material for: Direct laser writing of volumetric gradient index lenses and waveguides
Source: Light Sci Appl. 2020 Dec 3;9:196. doi: 10.1038/s41377-020-00431-3 (PMC7713360; doi:10.1038/s41377-020-00431-3)
Supplement: Supplementary file 1 — Supplementary Information [file 41377_2020_431_MOESM1_ESM.docx]

**Supplementary Information for:**

Direct Laser Writing of Volumetric Gradient Index Lenses and Waveguides

Christian R. Ocier^1,2,3,8^, Corey A. Richards^1,2,3,8^, Daniel A. Bacon-Brown^1,2,3^, Qing Ding^4^, Raman Kumar^4^, Tanner J. Garcia^2,3^, Jorik Van de Groep^5^, Jung-Hwan Song^5^, Austin J. Cyphersmith^6^, Andrew Rhode^1,2,3^, Andrea N. Perry^1,2,3^, Alexander J. Littlefield^4^, Jinlong Zhu^4^, Dajie Xie^1,2,3^, Haibo Gao^1,2,3^, Jonah F. Messinger^1,2,3^, Mark L. Brongersma^5^, Kimani C. Toussaint, Jr.^7^, Lynford L. Goddard^3,4*^, and Paul V. Braun^1,2,3,7*^

^1^Department of Materials Science and Engineering, University of Illinois at Urbana-Champaign, Urbana, IL, USA. ^2^Materials Research Laboratory, University of Illinois at Urbana-Champaign, Urbana, IL, USA. ^3^Beckman Institute for Advanced Science and Technology, University of Illinois at Urbana-Champaign, Urbana, IL, USA. ^4^Department of Electrical and Computer Engineering, University of Illinois at Urbana-Champaign, Urbana, IL, USA. ^5^Department of Materials Science and Engineering, Stanford University, Stanford, CA, USA. ^6^Carl R. Woese Institute for Genomic Biology, University of Illinois at Urbana-Champaign, Urbana, IL, USA. ^7^Department of Mechanical Science and Engineering, University of Illinois at Urbana-Champaign, Urbana, IL, USA. ^8^These authors contributed equally: Christian R. Ocier, Corey A. Richards. *email: lgoddard@illinois.edu, pbraun@illinois.edu

**Section 1: Scanning Electron Microscopy (SEM) Cross Section of Subsurface Hyperbolic Lens**

The cross section of a plano-convex hyperbolic lens printed in porous silicon (PSi) using SCRIBE was imaged using SEM (Fig. S1). The upper bound of the roughness was estimated to be <200 nm and was perhaps less.

**^
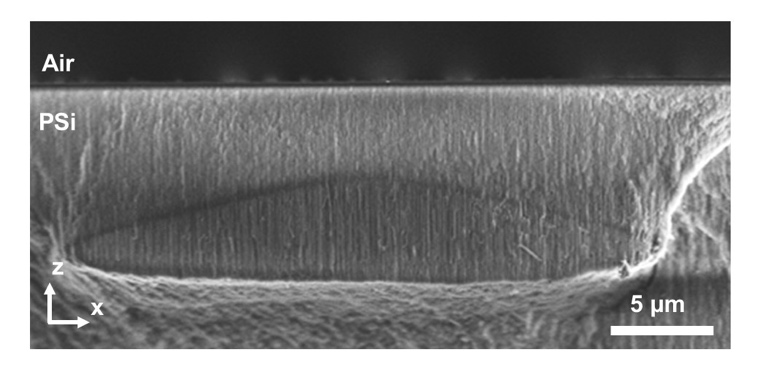
^**

**Fig. S1: Plano-convex hyperbolic lens printed within PSi.** SEM fractured cross-sectional image of a fracture surface of plano-convex hyperbolic lens printed within PSi.

**Section 2: Polymer Infilling Control**

SCRIBE can define regions of different polymer infilling, and thus different refractive index during a single writing process. As a demonstration, a checkerboard pattern was printed where adjacent squares were printed inside PSi with alternating laser powers of 7.5 and 15 mW. The fluorescence intensity of the resulting structure captured via multiphoton fluorescence microscopy is presented in Fig. S2.


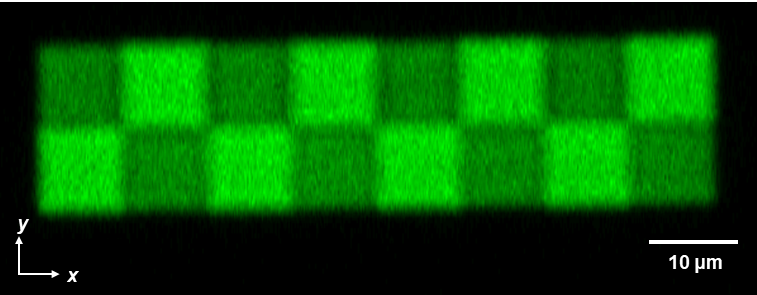


**Fig. S2:** **Fluorescence signal of regions of alternating polymer fill-fractions.** A checkerboard pattern was produced in PSi where adjacent squares were printed using alternating average laser powers of 7.5 and 15 mW. The alternating fluorescence intensities demonstrate that the degree of polymerization (and by extension the effective refractive index) can be spatially tuned in a single writing process.

**Section 3: Effect of Birefringence on Biprism Fringe Spacing**

It is necessary to verify the accuracy of Equation (1) in the main text. However, Equation (1) assumes an isotropic refractive index, but PSi is a highly birefringent material. While the birefringence of PSi can be easily measured using ellipsometry, measuring the birefringence of the polymer/PSi composite is not straightforward. Polymerized regions may be stitched together for ellipsometry, but the stitching errors over millimetre-scale regions can make ellipsometry unreliable. The effect of birefringence may be mitigated if the angle of refraction inside the prism is small. To verify this assumption, the interference pattern produced by a simulated isotropic prism was compared to those produced by simulated birefringent prisms, where the ordinary refractive index (*n_o_*) was held constant (Fig. S3a).

The measured fringe spacings for four featured simulations are shown in Fig. S3b-c. The difference between the fringe spacings is small between each sample, meaning that the extraordinary refractive index (*n_e_*) does not significantly affect the produced interference pattern. As shown in Fig. S3c, *n_o_* was extracted once the measured *d_fringes_* and *α* were plugged into Equation (1) of the main text. Equation (1) effectively gave the ordinary refractive index and was accurate for all optical elements presented in this article since the angle of refraction was always reasonably small. As a final verification that *n_o_* was the primary determinant of the fringe spacing, prisms with constant *n_e_* but varying *n_o_* were simulated as shown in Fig. S4a. The overlaid intensities of the interference patterns in Fig. S4b show that *d_fringes_* changed drastically as *n_o_* was altered. Once again, *n_o_* was extracted when using Equation (1) as graphically displayed in Fig. S4c.


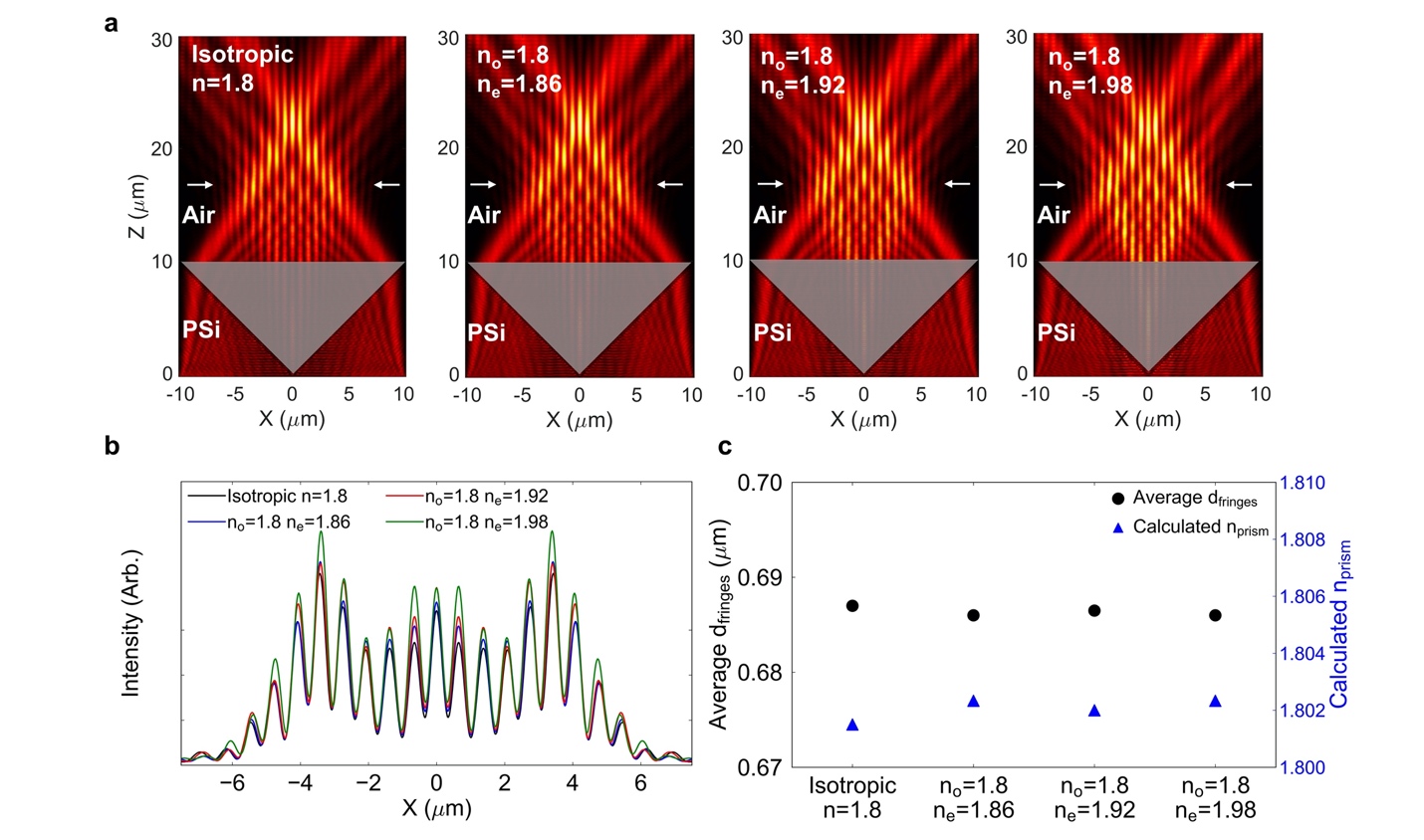


**Fig. S3:** **Biprism simulations with constant n_o_. a** Simulated interference patterns produced by biprisms with α=45° refracting 633 nm light that was polarized along the x-axis. The ordinary refractive index was held constant, while the extraordinary refractive index was increased from left to right. **b** Overlaid intensities of the interference patterns produced by the prims in **a**. The intensities were measured between the arrows shown in **a**. **c** Average spacing of the fringes in the interference patterns (left axis) and corresponding refractive index calculated from the fringe spacing using Equation (1) in the main text (right axis). The calculated values of n_prism_ were roughly equal to the simulated value of n_o_, which was 1.8, suggesting that n_e_ had little effect on the fringe spacing of the output interference pattern.


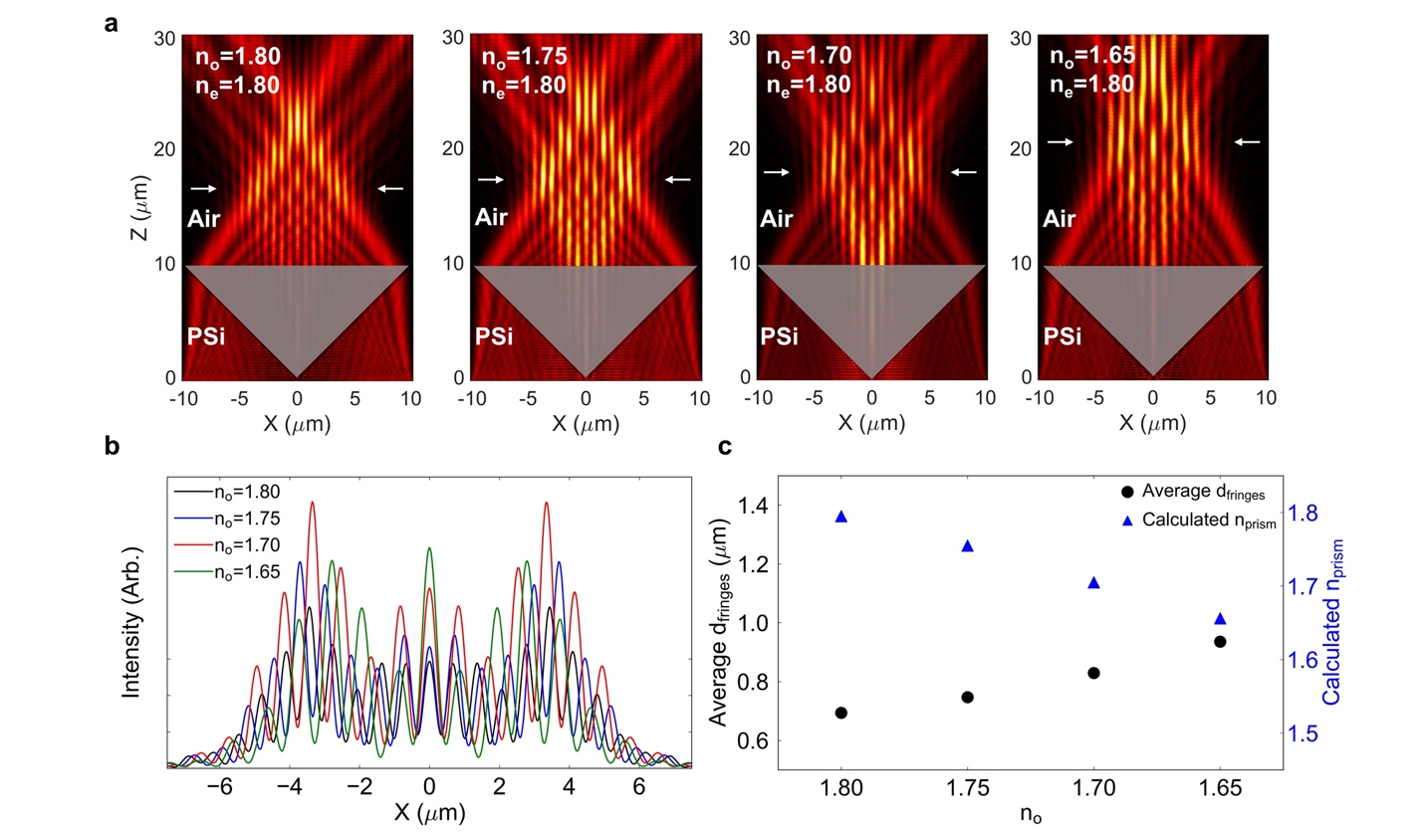


**Fig. S4:** **Biprism simulations with constant n_e_. a** Simulated interference patterns produced by biprisms with α=45° refracting 633 nm light that was polarized along the x-axis. The extraordinary refractive index was held constant, while the ordinary refractive index was decreased from left to right. **b** Overlaid intensities of the interference patterns produced by the prims in **a**. The intensities were measured between the arrows shown in **a**. **c** Average spacing of the fringes in the interference patterns (left axis) and corresponding refractive index calculated from the fringe spacing using Equation (1) in the main text (right axis). The calculated values of *n_prism_* were always roughly equal to the simulated value of *n_o_*, regardless of its value, once again suggesting that *n_e_* had little effect on the fringe spacing of the output interference pattern.

**Section 4: The Effect of Exposure on Geometry Fidelity**

Underexposure in SCRIBE manifested itself as poorly defined geometries and occurred when the laser exposure within the polymer-filled scaffold was low. However, small amounts of photoresist can still be minimally polymerized within the scaffold, leading to minor changes in refractive index. An example of this is the barely perceivable, underexposed prism in Fig. S5a printed in porous silicon dioxide (PSiO_2_) with a laser power of 5 mW and a scan speed of 10 mm s^-1^. Although quantifying the optical constants of these minimally polymerized regions is difficult, these low-density voxels are critical for accessing broad index ranges needed in certain gradient refractive index (GRIN) optics. For example, a Luneburg lens requires an index contrast of ~0.41, exceeding the ~0.3 index range shown in Fig. 4 of the main text. To reproduce the full index profile of the Luneburg lens, the measurable refractive index values were extrapolated to the low exposure index regime.

Fig. S5b shows a prism with the proper geometric dimensions formed under intermediate exposure conditions, which span the range from above the low exposure regime up to the threshold power where damage occurs (see damaged prism in Fig. S5c). The laser powers and exposure conditions at which either extreme occurred depended on the scan speed applied during printing. For the prisms shown in Fig. 4d and 4e of the main text, the upper and lower bounds of the refractive index curves mark the range of powers where the prism geometries can be reliably reproduced.


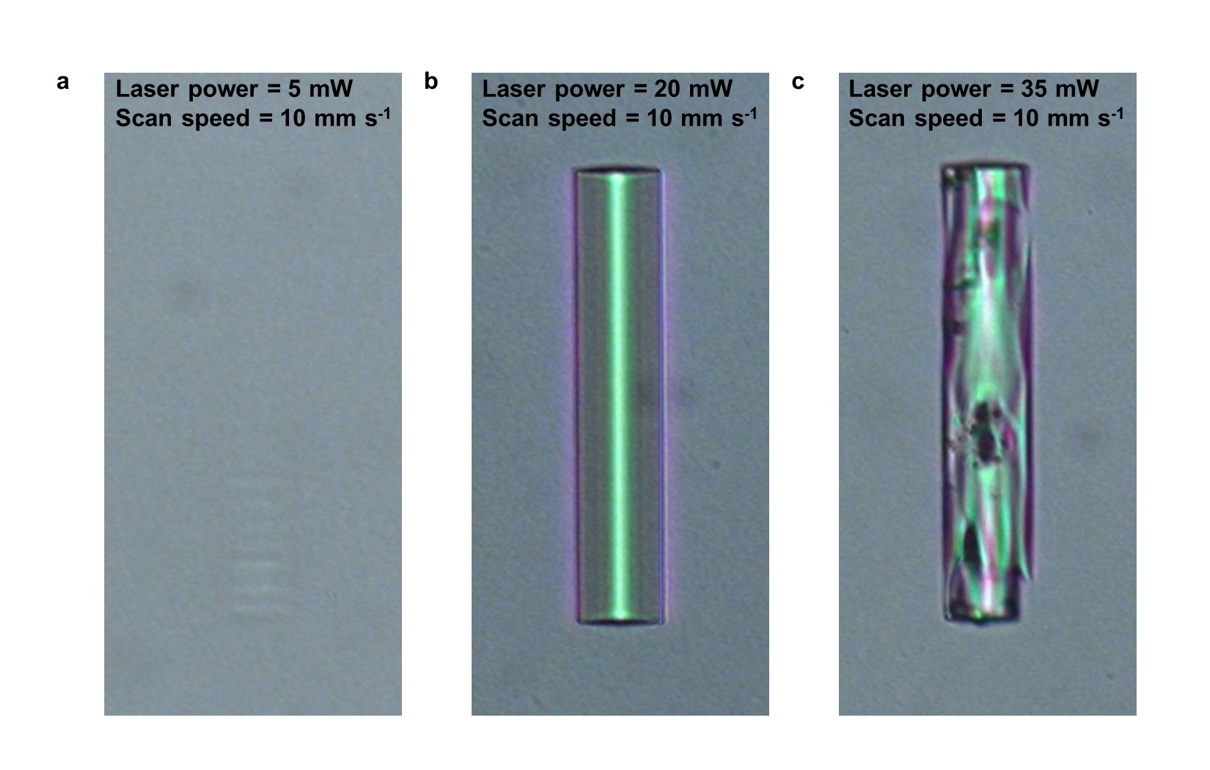


**Fig. S5: Effect of exposure on printed structures.** Top view transmission optical images of prisms printed in PSiO_2_ scaffolds under low laser exposure **(a)**, intermediate laser exposure **(b)**, and high laser exposure **(c)**. The prisms are 20 µm wide and 100 µm long.

**Section 5: Photonic Nanojet Generators**

Dielectric microspheres and microcylinders have been used to generate photonic nanojets (PNJs)^1,2^. PNJ generators that consist of a cascade of integrated multimode waveguide based microstructures were proposed^3^ and analysed using eigenmode expansion and transmission matrix theory^4^. Fig. S6a shows the device schematic. Each microstructure consists of a waveguide supporting stage and a cap. By controlling the length and width of each waveguide and the radius of curvature of each cap, the wavefront profile at various locations can be engineered through the use of modal interference. This enables suppression of the strength of the side lobes, resulting in a cleaner PNJ. However, assembling and aligning these individual free objects is challenging, limiting their scalability and range of applications. As we show here, SCRIBE offers a unique opportunity to fabricate this structure.

Fig. S6b shows the optical multiphoton fluorescence intensity image of a typical SCRIBE fabricated two-element cascaded device. The device was written in PSi with a design index of 1.6 at 633 nm. Each waveguide had a width of 6 μm along *x* and was extended along *y*. The length of the bottom waveguide was 3 μm; however, the length of the top waveguide (*l*) was set to 2.3 μm to ensure the top cap, a hemicylinder of radius *r*=3 µm, was located exactly within the energy convergence region^3^ to produce a PNJ that was narrow in *x* and *y* but elongated in *z* and located outside the PSi film. For this device Δ*l*=0 µm and the bottom cap was also a hemicylinder of radius *r*=3 μm. Thus, the device measured 11.3 μm along *z*.

Fig. S6c shows the simulated and experimentally measured intensity profiles in the *xz*-plane of a device that only had the bottom microstructure (*r*=3 µm). Fig. S6d shows the corresponding images for the cascaded device. Both devices extended 100 μm in *y*. Due to modal interference, the single microstructure showed several very strong side lobes in the air region. For the cascaded device (*r*=3 μm, Δ*l*=0 μm, *l*=2.3 μm), the linear combination of modes exiting the bottom microstructure excited specific modes in the top waveguide, whose length was chosen to control modal evolution and therefore the field profile upon exiting the device. Consequently, a cleaner PNJ hotspot was obtained both in simulation and experiment.


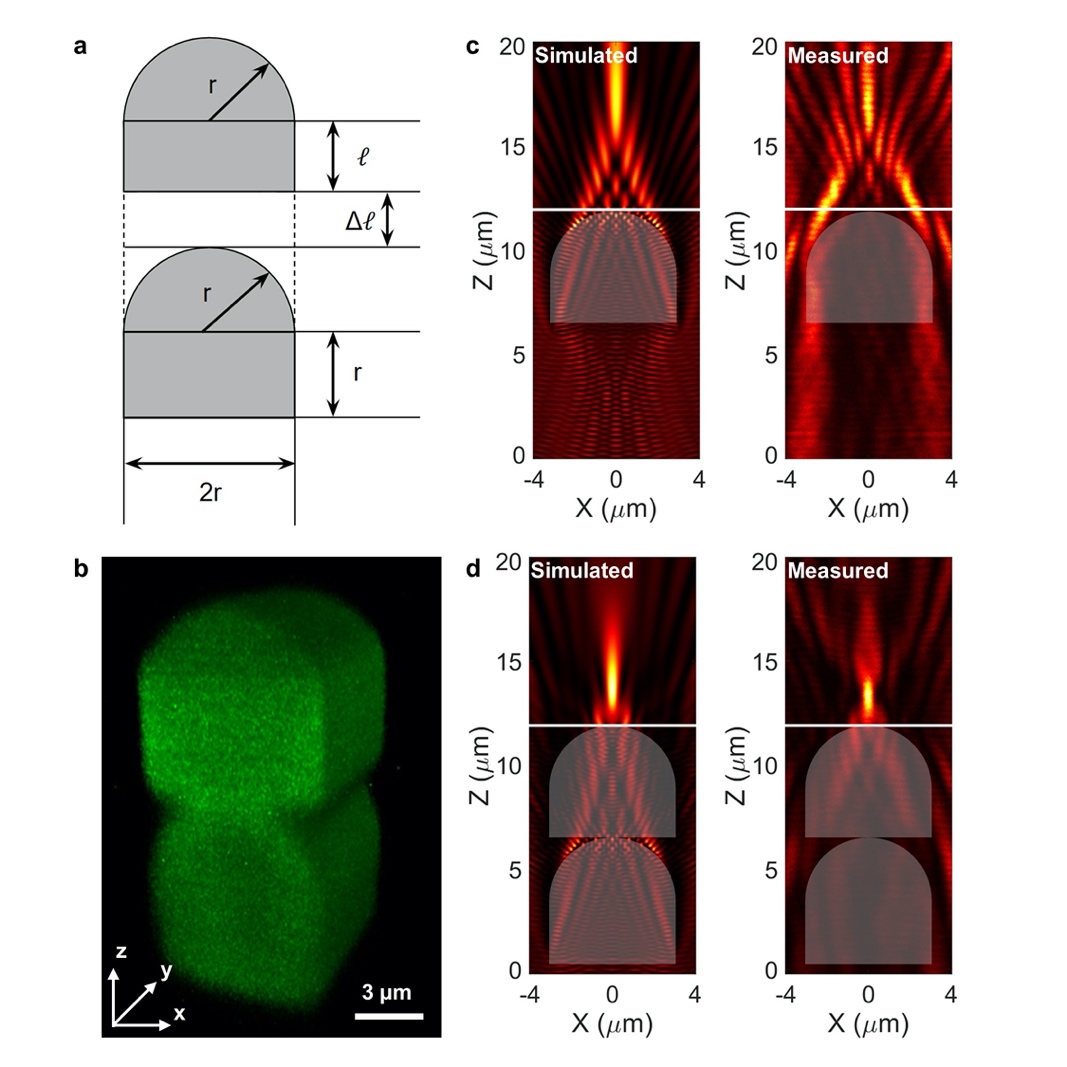


**Fig. S6: Cascaded waveguide PNJ generators. a** Device schematic (variables defined in text). **b** Multiphoton image of a cascaded capped waveguide PNJ generator written inside PSi. **c, d** Simulated and experimentally measured focusing profiles at 633 nm of a (**c**) single and (**d**) double microstructure. Dimensions described in text.

**Section 6: GRIN Lens Top Views**

GRIN axicon and the Luneburg lens were imaged from the top (Fig. S7) to characterize their focusing behaviour.


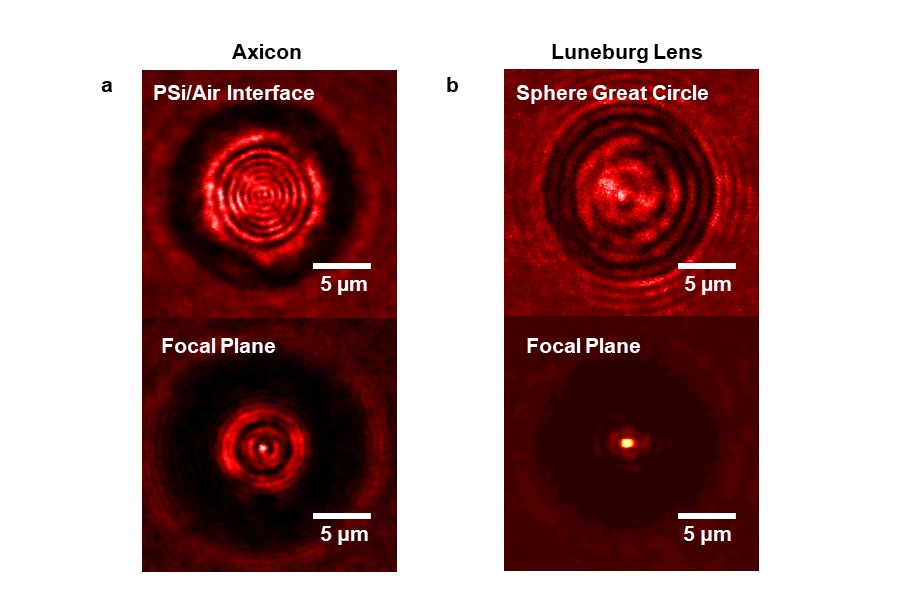


**Fig. S7: GRIN optical elements in PSi. a,** Measured *xy*-plane intensity profile of a GRIN axicon at the PSi/Air interface (top), and in focus at the central Bessel beam (bottom). **b,** Measured *xy*-plane intensity profile of a 3D Luneburg lens at the central great circle (top), and in focus immediately above the PSi/air interface (bottom).

**Section 7: Design of Additional Planar Gradient Index Lenses**

SCRIBE lithography can generate planar lenses that focus light due to their radial GRIN profiles. GRIN parabolic lenses (Fig. S8) and GRIN cubic Airy beam phase masks (Fig. S9) are two planar optics not highlighted in the main text. These flat lenses were formed by altering *n(r)* from Equation (2) in the main text. For parabolic lenses:

$$\begin{aligned} n\left( r \right)=n_{centre}-\frac{n_{centre}-n_{edge}}{R_{lens}^{2}}*r^{2} \#\left( S1 \right) \end{aligned}$$

Similarly, GRIN Airy beam phase masks are fabricated with a GRIN profile according to:

$$\begin{aligned} n\left( r \right)=\frac{\left( \left( n_{max}-n_{min} \right)\left( r-\frac{R_{lens}}{2} \right)\left( \left( \frac{R_{lens}}{2} \right)^{2}-8*\frac{R_{lens}}{2}*r+4*r^{2} \right) \right)}{2*\left( \frac{R_{lens}}{2} \right)^{3}}+n_{mid} \#\left( S2 \right) \end{aligned}$$

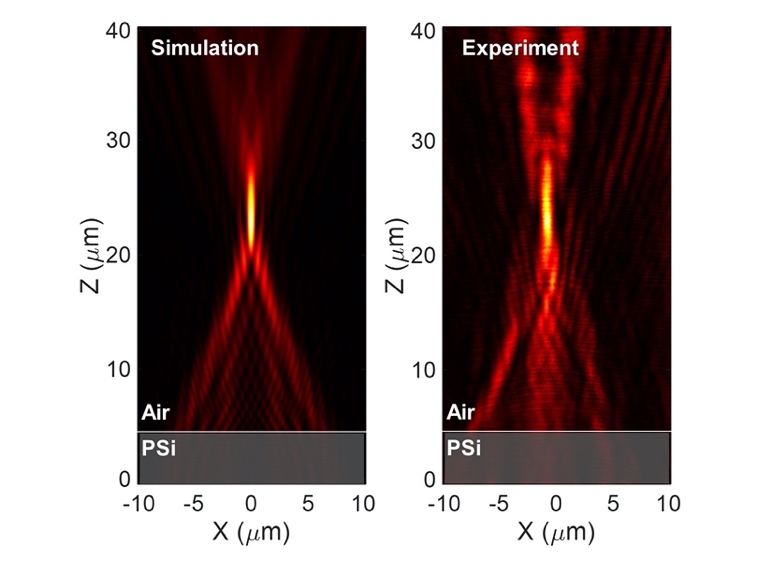


**Fig. S8:** **Planar GRIN parabolic lens.** A GRIN parabolic lens was printed inside PSi. The measured focal behaviour agreed strongly with simulation, adding to the roster of GRIN lenses that can be fabricated with SCRIBE.


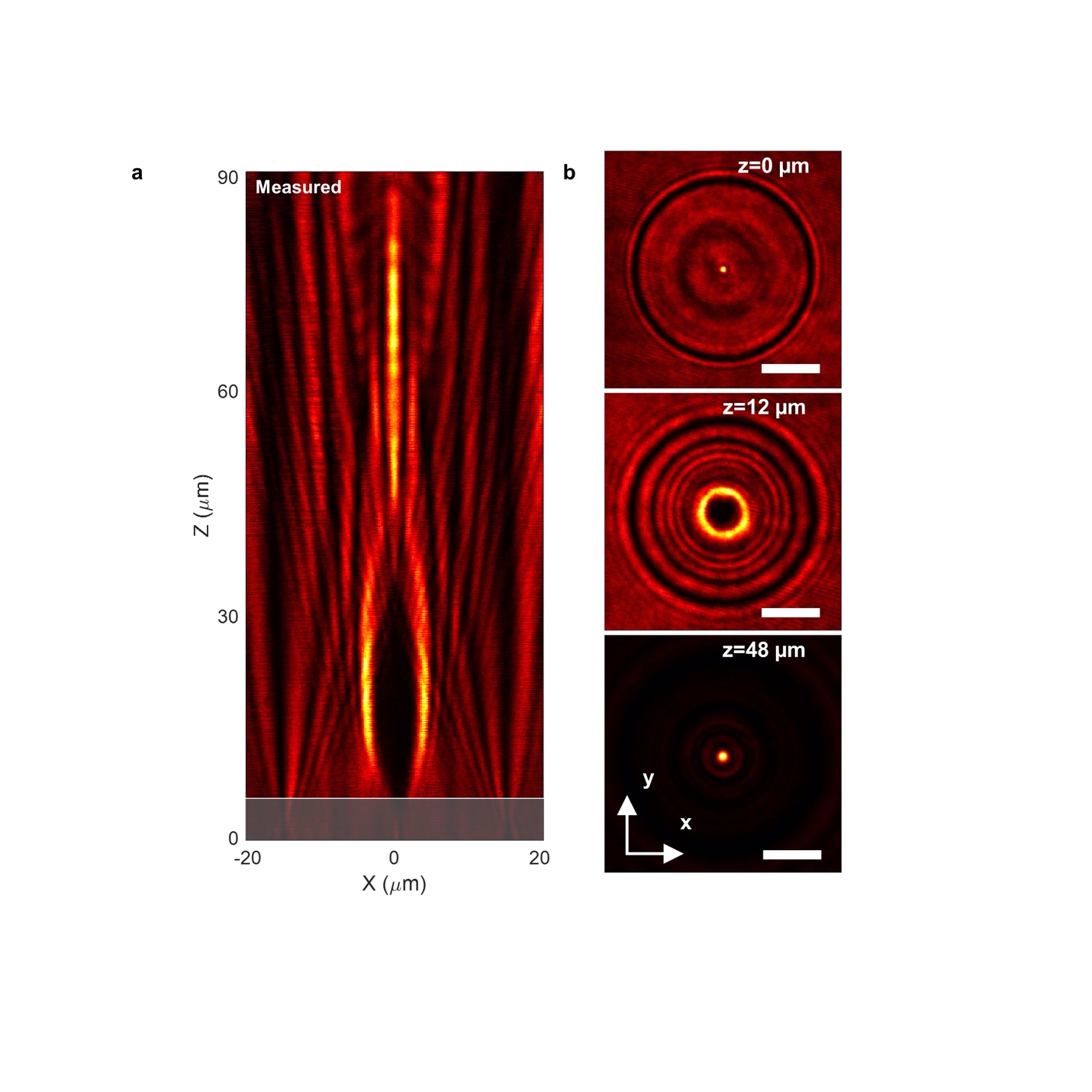


**Fig. S9:** **Planar GRIN Airy beam generating phase mask. a** *x-z* intensity scan of an Airy beam formed by a GRIN cubic phase mask. The main intensity lobes propagated along curved paths and formed a Bessel-like beam at the focal spot. A region of low intensity was formed between the two main lobes. **b** Top view images taken at different heights above the planar Airy beam phase mask. The region of low intensity was seen at the centre of the phase mask 12 μm above the surface. The beam collapsed into focus at 48 μm. Scale bars are 10 µm.

**Section 8: Demonstration of 1-µm Diameter Waveguides**

The point spread function (PSF) of voxels written with SCRIBE necessitates a different writing approach for fabricating small (< 5 μm) optical elements. For 1 μm diameter waveguides, it is not possible to directly write a cylindrical cross section because of the asymmetry of the voxel. Instead, writing symmetric micron-scale features requires the exploitation of the proximity effect to shape more symmetric objects of this size. In multiphoton polymerization, the proximity effect is crosstalk between voxels written close to one another, resulting in a final geometry that differs from what is expected when considering the PSF of isolated voxels. Fig. S10 shows schematics and SEM images for fabricating both single index and GRIN waveguides using SCRIBE. Instead of creating a 1 μm cylindrical stereolithography file (e.g., using AutoCAD) to render the structure, the proximity effect was utilized to form waveguides with a circular cross section by writing multiple line voxels that were stepped laterally in *x*. The schematic in Fig. S10a shows how this is accomplished by spacing seven line voxels written in *y* at a scan speed of 10 mm s^-1^ with 100 nm steps in *x* between each voxel, at an average laser power of 25 mW and using the proximity effect to generate a circular profile. Fig. S10b shows the SEM cross section of the fabricated waveguide. Subsurface waveguides coupled to ring resonators were also fabricated using this method in addition to the devices featured in Fig. 7 of the main text.

Refractive index variations in a waveguide’s cross section can be imparted in SCRIBE by changing the laser exposure for each of line voxel, as shown in Fig. S10 c-d. This GRIN waveguide was made by spacing seven line voxels in x with a spacing of 100 nm, as depicted by the schematic in Fig. S10c. The centre voxel was exposed at an average laser power of 27.5 mW, while the pairs of voxels decaying away from the centre were exposed at decreasing increments of 2.5 mW each iteration, yielding a core that has a higher refractive index. The cylindrical cross section imaged in Fig. S10d showed a nearly symmetric profile. Waveguides with this GRIN profile were used for the measurements in the main waveguides section of this work (Fig. 7).


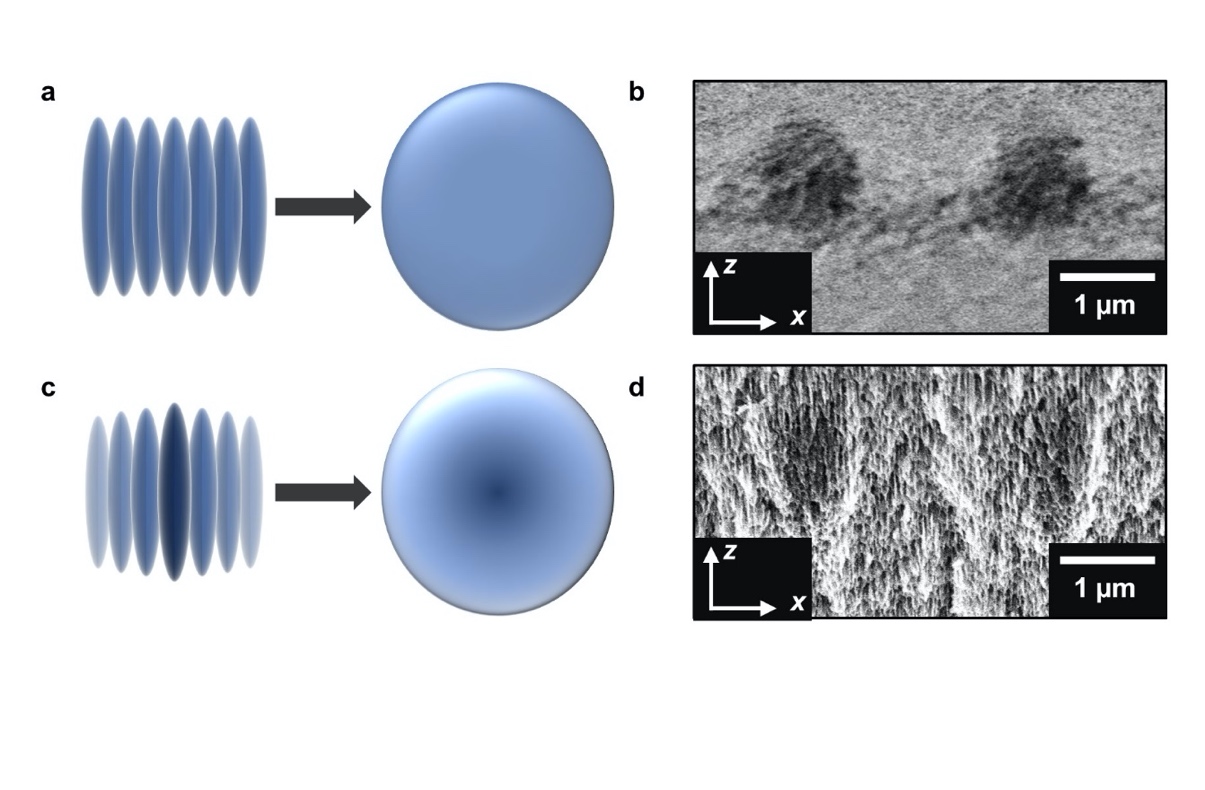


**Fig. S10: Symmetric waveguide design. a,** Schematic depicting the fabrication of a 1 μm diameter single index waveguide by spacing seven line voxels together. When seven line voxels were written together in this way, the final waveguide cross section was symmetric due to the proximity effect. The spacing between each line voxel was 100 nm. **b,** SEM fractured cross section of the line voxel design used to form single index subsurface waveguides. The cross section of this designed waveguide measured 1.09 ± 0.07 μm in X and 1.11 ± 0.12 μm in Z, with error attributed to random fluctuations that occur during the writing process. **c,** Schematic depicting the fabrication of a subsurface GRIN waveguide by symmetrically altering the refractive index across seven line voxels, with the highest average laser power at the centre. The spacing between each line voxel was 100 nm. **d,** SEM fracture cross section of the voxel design used to form GRIN subsurface waveguides. The cross section of this designed voxel measured 1.10 ± 0.12 μm in X and 1.21 ± 0.08 μm in Z.

The measured loss for these single index waveguides in TE mode ranged from 3.08 dB mm^-1^ to 3.54 dB mm^-1^ with an average value of 3.34 dB mm^-1^. The transmission measurements for the single index waveguides in TE mode are plotted in Fig. S11a and Fig. S11b. Fig. S12 compares the free spectral range (FSR) and Q-factor values extracted from the spectra of the GRIN and single index waveguides in TE mode. The FSR values for the GRIN waveguides imply that the group indices for the TE mode were 1.584, 1.556, and 1.557 for the overcoupled (400 nm gap), critically coupled (600 nm gap), and the undercoupled (800 nm gap) waveguides, respectively, as described in Fig. S12a and Fig. S12c, while the four single index waveguides shown in Fig. S12b and Fig. S12d had a group index of 1.428, 1.427, 1.434, and 1.434, respectively. The larger group index of the GRIN waveguides likely originated from the higher average laser power used to expose the centre portion of the core of the GRIN waveguides, leading to the higher Q-factors observed in the GRIN waveguides of Fig. S12c compared to the single index waveguides of Fig. S12d. Results for the TM mode were similar to those for TE. The group indices for the GRIN waveguides were 1.556, 1.551, and 1.546 for the 400 nm, 600 nm, and 800 nm gaps, respectively. The group indices for the four single index waveguides were 1.444, 1.440, 1.442, and 1.443, respectively. Overall, the group indices for TE and TM agreed to about 1-2%, which is comparable to the limits of our measurement. This level of agreement indicates that the birefringence effects are negligible in PSiO_2_.

**
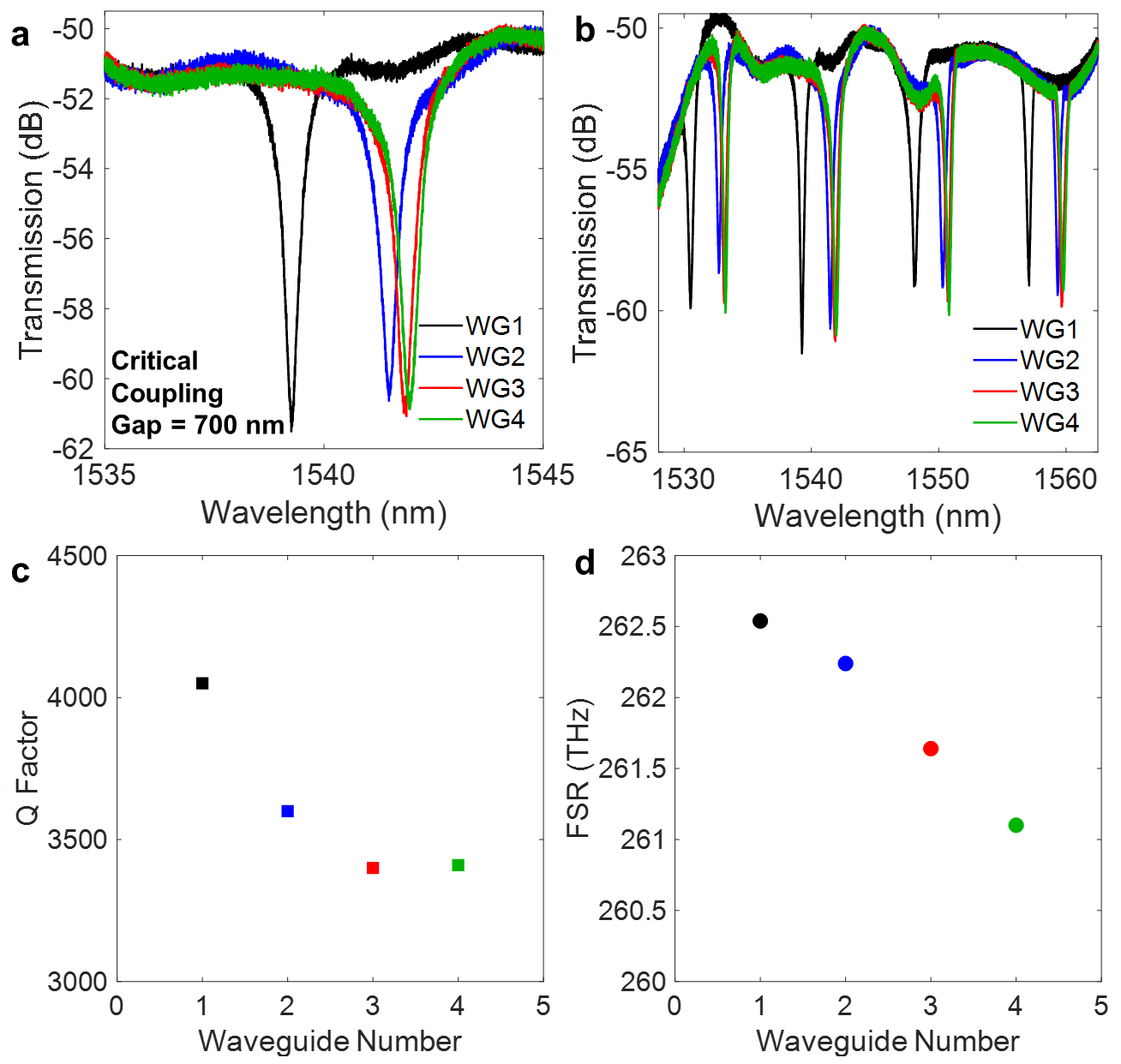
**

**Fig. S11:** **Transmission spectra from single index subsurface waveguides with circular cross section for the TE mode. a,** Zoomed in region of experimentally measured microring resonator spectra for four different 1 μm diameter single index waveguide devices written at an average laser power of 25 mW. **b,** Full wavelength range microring resonator spectra for the same devices as in **a**.


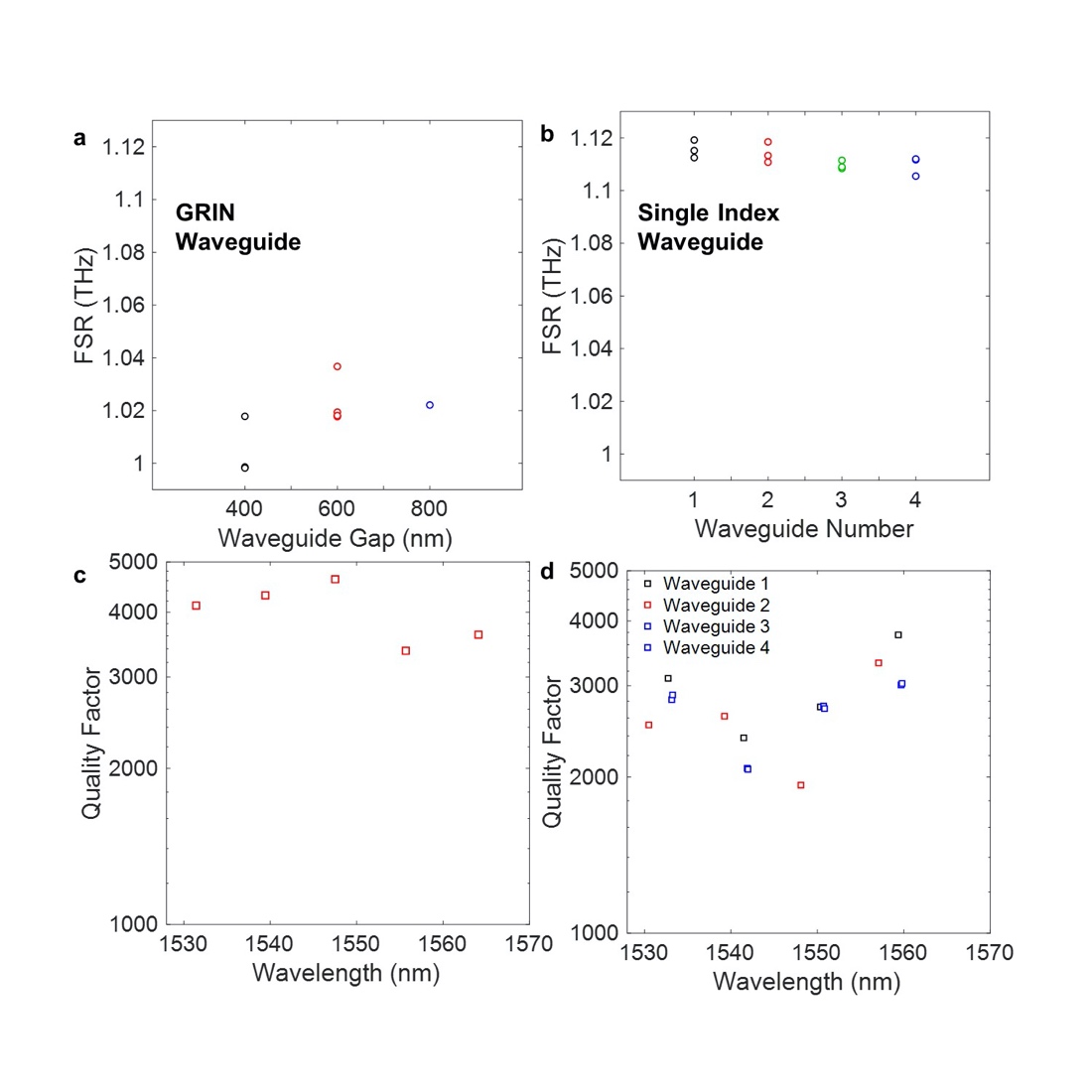


**Fig. S12:** **Calculated FSR and Q-factors for GRIN and single index waveguides for the TE mode. a,** FSR for the experimentally measured resonance dips in GRIN waveguides with an overcoupled (400 nm, black), critically coupled (600 nm, red), and undercoupled (800 nm, blue) resonator/waveguide gap. **b,** FSR for the experimentally measured resonances in single index waveguides. **c,** Q-factors calculated for a critically coupled GRIN microring (gap = 600 nm) for the five different resonances. **d,** Q-factors calculated for four critically coupled single index waveguides for the four different resonances.

**Section 9: Ring Resonator Loss Analysis**

Ring resonators can be coupled to waveguides to measure optical propagation and bending losses. Because the loss is extracted from resonance characteristics such as full width at half maximum (FWHM), FSR, and extinction ratio (ratio of maximum to minimum transmitted intensity), it is independent of the reflectivity of the bus waveguide facets and the fibre-to-waveguide coupling losses. The two chief parameters of a ring resonator are: the transmission coefficient of the directional coupler, $t_{d}$, and the roundtrip loss coefficient $\alpha_{rt}$ going around the ring. The most common approach to extract $t_{d}$ and $\alpha_{rt}$ is to curve fit a single resonance with the ring resonator transmission equation. This approach assumes that both $t_{d}$ and $\alpha_{rt}$ are of comparable magnitude and consequently, these parameters cannot be detangled unambiguously from a single resonance because they enter the equation symmetrically. The FSR ($\Delta\lambda_{FSR}$), FWHM ($\Delta\lambda_{FWHM}$), and extinction ratio ($\xi$) were extracted for all the measured resonances in the spectrum. The parameters $t_{d}$ and $\alpha_{rt}$ were then computed using Equation (S3)^5^:

$$\begin{aligned} \left( t_{d},\alpha_{rt} \right)=\left( \frac{A}{B} \right)^{\frac{1}{2}}\pm\left( \frac{A}{B}-A \right)^{\frac{1}{2}}\#\left( S3 \right), \end{aligned}$$

where the variables A and B are defined as

$$\begin{aligned} A=\frac{\cos\left( \frac{\pi}{F} \right)}{1+sin\left( \frac{\pi}{F} \right)}\#\left( S4 \right) \end{aligned}$$

$$\begin{aligned} B=1-\left[ \frac{1-\cos\left( \frac{\pi}{F} \right)}{1+cos\left( \frac{\pi}{F} \right)} \right]\frac{1}{\xi}\#\left( S5 \right) \end{aligned}.$$

The parameter $F$ is the finesse defined as

$$\begin{aligned} F=\frac{\Delta\lambda_{FSR}}{\Delta\lambda_{FWHM}}\#\left( S6 \right) \end{aligned}$$

and the extinction ratio is defined as

$$\begin{aligned} \xi=\frac{Maximum Transmitted Intensity}{Minimum Transmitted Intensity}=\frac{T_{max}}{T_{min}}\#\left( S7 \right) \end{aligned}.$$

In general, the parameters $t_{d}$ and $\alpha_{rt}$ exhibit different dependencies on wavelength. The transmission coefficient, $t_{d}$, varies sinusoidally with wavelength whereas the loss coefficient, $\alpha_{rt},$ remains relatively constant. By analysing multiple resonances, this behaviour can be observed, and $t_{d}$ and $\alpha_{rt}$ can be unambiguously detangled from Equation (S3).

After the transmission coefficient and loss coefficient were extracted for the microring resonances measured in the C-band (i.e., near 1550 nm wavelength), the loss value was determined by:

$$\begin{aligned} \alpha_{dB per mm}=\frac{10{log}_{10} \left( \alpha_{rt} \right)}{Circumference of Ring in mm}\#\left( S8 \right). \end{aligned}$$

**Section 10: Low Absorption Scaffolding Materials**

It is necessary to use a porous scaffolding material that is reasonably transparent at the writing laser’s wavelength when using SCRIBE lithography. The wavelength of the Nanoscribe’s titanium:sapphire writing laser is 780 nm. It is known that PSiO_2_ is transparent across the visible and near-IR. Using ellipsometry, we found that our PSi also exhibited low absorption (~15 cm^-1^) at 780 nm (Fig. S13), which corresponds to a transmission of >96% at a depth of 25 µm. At this point, we have not determined the maximum printing depth; the deepest structure printed inside PSi was the achromat, Fig. 5 of the paper, which was 19.5 microns below the PSi surface at its deepest point. Based on absorption alone, we expect it is possible to print to depths of >50 µm inside PSi. We did not attempt such an experiment and cannot anticipate the effects of scattering or heating.

**
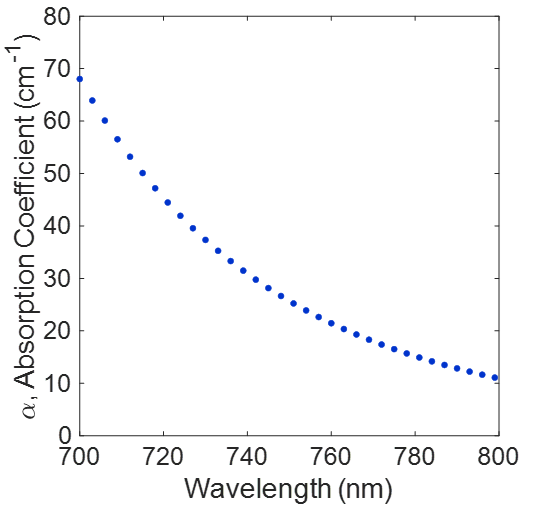
**

**Fig. S13:** **Absorption coefficient of porous silicon.** The extinction coefficient (k) of PSi was measured from 700 to 800 nm using ellipsometry (see methods section of the main text). The absorption coefficient, plotted here, is given by α=4πk/λ.

**References:**

1. Lu, Y. F., Zhang, L., Song, W. D., Zheng, Y. W. & Luk’yanchuk, B. S. Laser writing of a subwavelength structure on silicon (100) surfaces with particle-enhanced optical irradiation. *Journal of Experimental and Theoretical Physics Letters* **72**, 457–459 (2000).

2. Chen, Z., Taflove, A. & Backman, V. Photonic nanojet enhancement of backscattering of light by nanoparticles: a potential novel visible-light ultramicroscopy technique. *Optics Express* **12**, 1214–1220 (2004).

3. Zhu, J. & Goddard, L. L. Spatial control of photonic nanojets. *Optics Express* **24**, 30444–30464 (2016).

4. Zhu, J. & Goddard, L. L. Controlling Photonic Nanojets: From the Standpoint of Eigenmodes. *IEEE Photonics Technology Letters* **30**, 75–78 (2018).

5. McKinnon, W. R. *et al.* Extracting coupling and loss coefficients from a ring resonator. *Optics Express* **17**, 18971–18982 (2009).
